# Supplementary material for: Modeling Effects of Variable preBötzinger Complex Network Topology and Cellular Properties on Opioid-Induced Respiratory Depression and Recovery
Source: eNeuro. 2024 Mar 1;11(3):ENEURO.0284-23.2023. doi: 10.1523/ENEURO.0284-23.2023 (PMC10921262; doi:10.1523/ENEURO.0284-23.2023)
Supplement: Code files — Download Code files, ZIP file. [file eneuro-11-ENEURO.0284-23.2023-s001.zip › prebot-opioid-model-main/figure_notebooks/fig6and7/fig6/fig6_gnap_phase_diagrams.pdf]

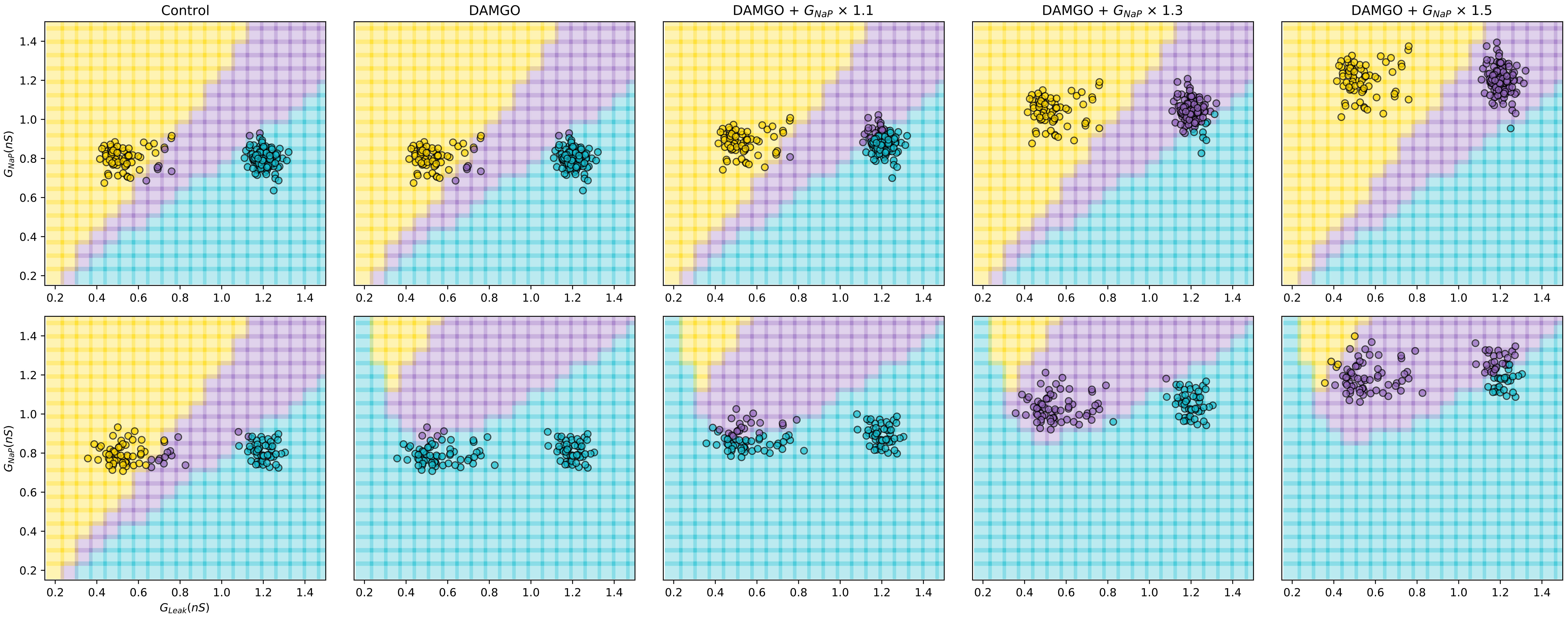
Control
DAMGO
DAMGO + G_{NaP} × 1.1
DAMGO + G_{NaP} × 1.3
DAMGO + G_{NaP} × 1.5
$G_{NaP}(nS)$
$G_{Leak}(nS)$
0.2
0.4
0.6
0.8
1.0
1.2
1.4
