# Supplementary figures and images for: Modeling Effects of Variable preBötzinger Complex Network Topology and Cellular Properties on Opioid-Induced Respiratory Depression and Recovery
Source: eNeuro. 2024 Mar 1;11(3):ENEURO.0284-23.2023. doi: 10.1523/ENEURO.0284-23.2023 (PMC10921262; doi:10.1523/ENEURO.0284-23.2023)

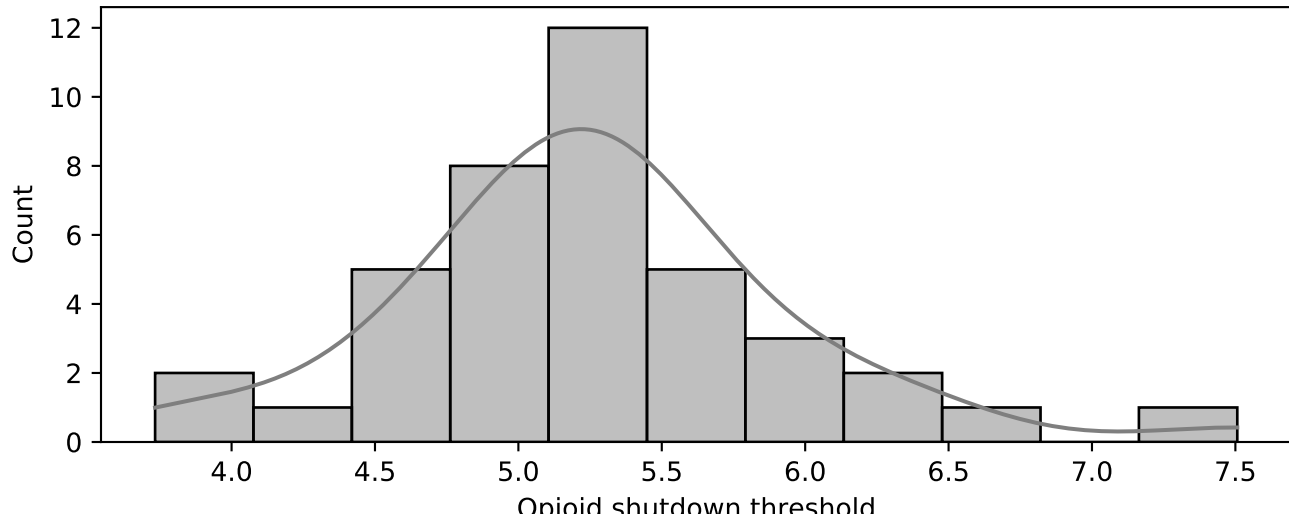

Supplement: Code files — Download Code files, ZIP file. [file eneuro-11-ENEURO.0284-23.2023-s001.zip › prebot-opioid-model-main/figure_notebooks/fig1/fig1_hist.pdf]

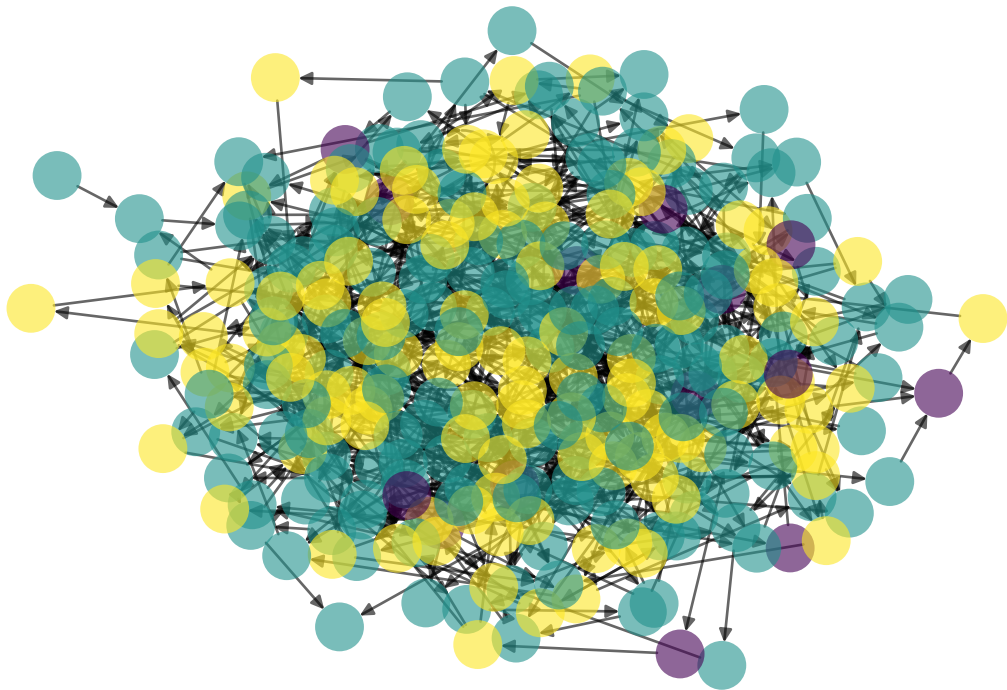

Supplement: Code files — Download Code files, ZIP file. [file eneuro-11-ENEURO.0284-23.2023-s001.zip › prebot-opioid-model-main/figure_notebooks/fig1/fig1_networkx_graph.pdf]

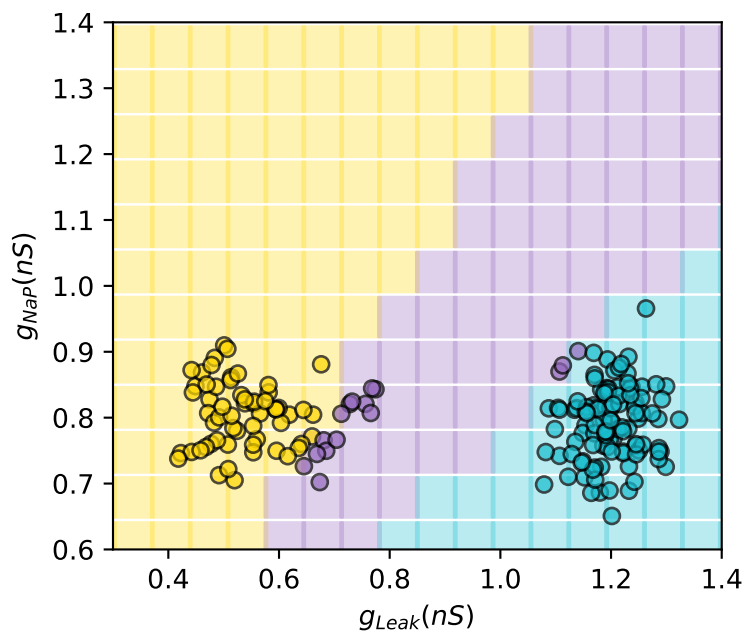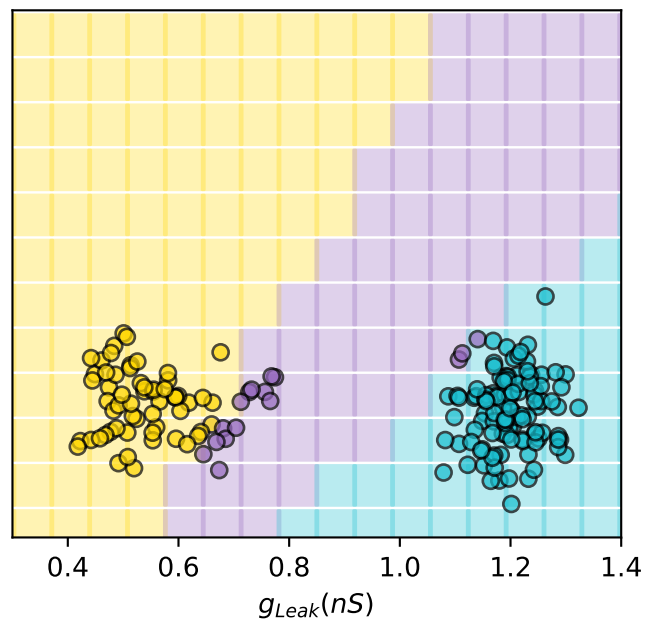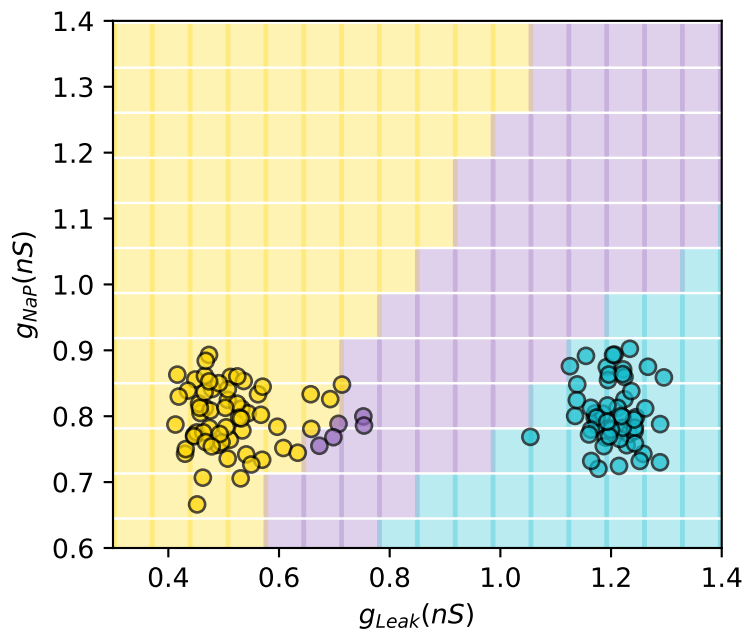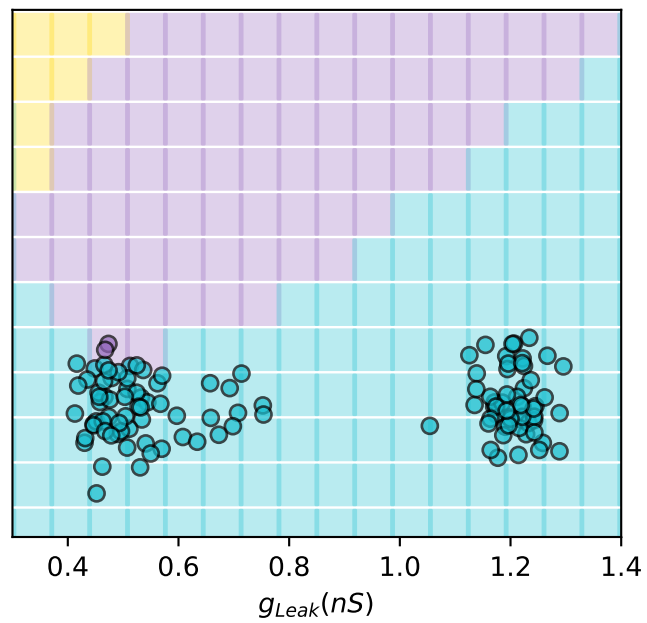

Supplement: Code files — Download Code files, ZIP file. [file eneuro-11-ENEURO.0284-23.2023-s001.zip › prebot-opioid-model-main/figure_notebooks/fig1/fig1_phase_diagrams.pdf]

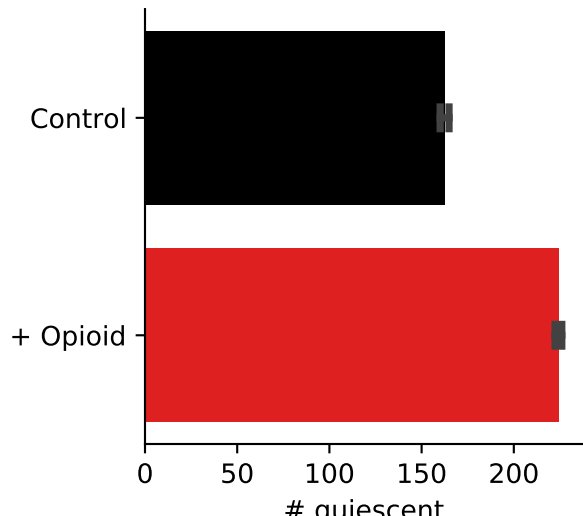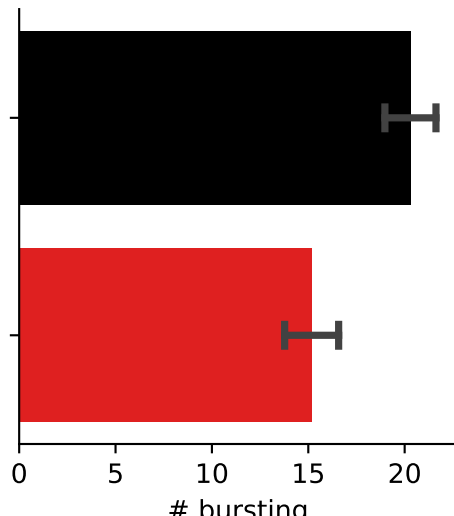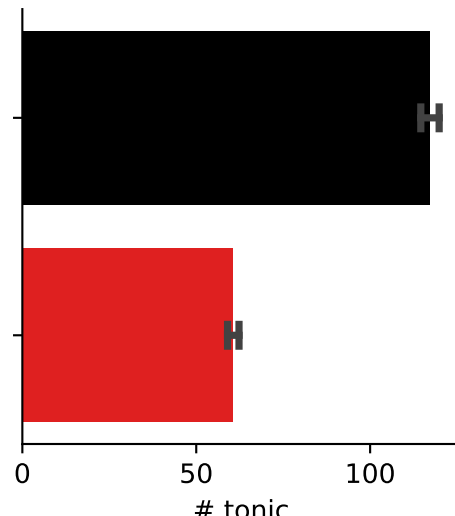

Supplement: Code files — Download Code files, ZIP file. [file eneuro-11-ENEURO.0284-23.2023-s001.zip › prebot-opioid-model-main/figure_notebooks/fig1/fig1_tbq_barplots.pdf]

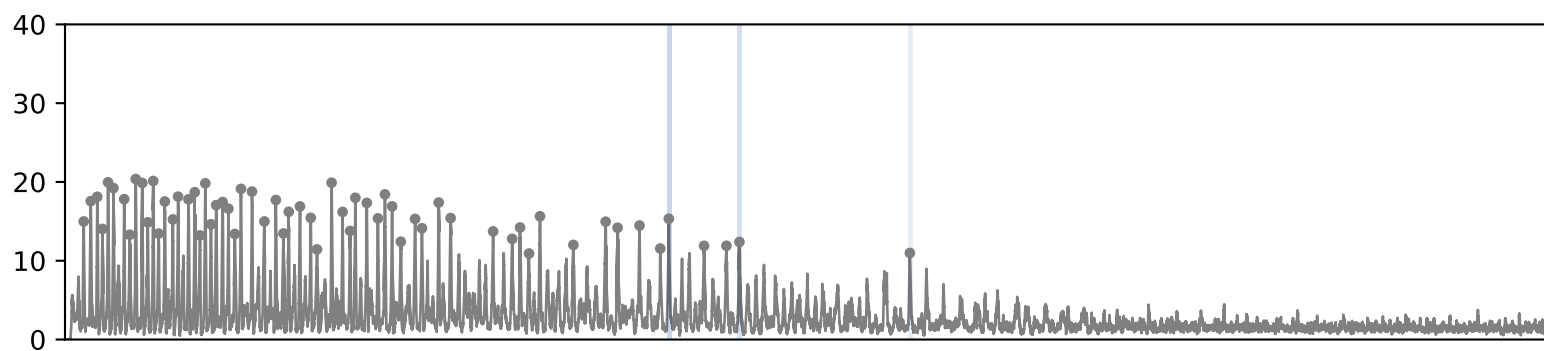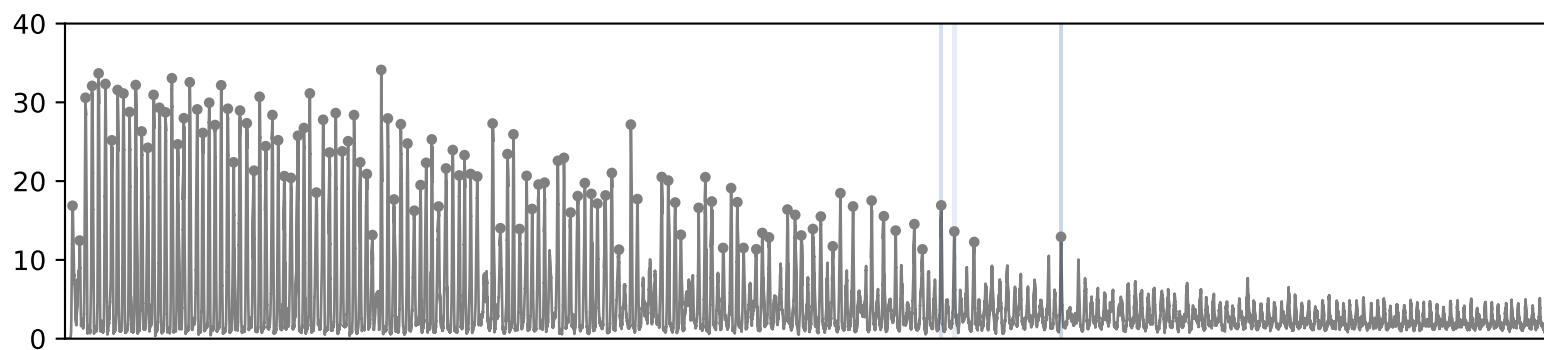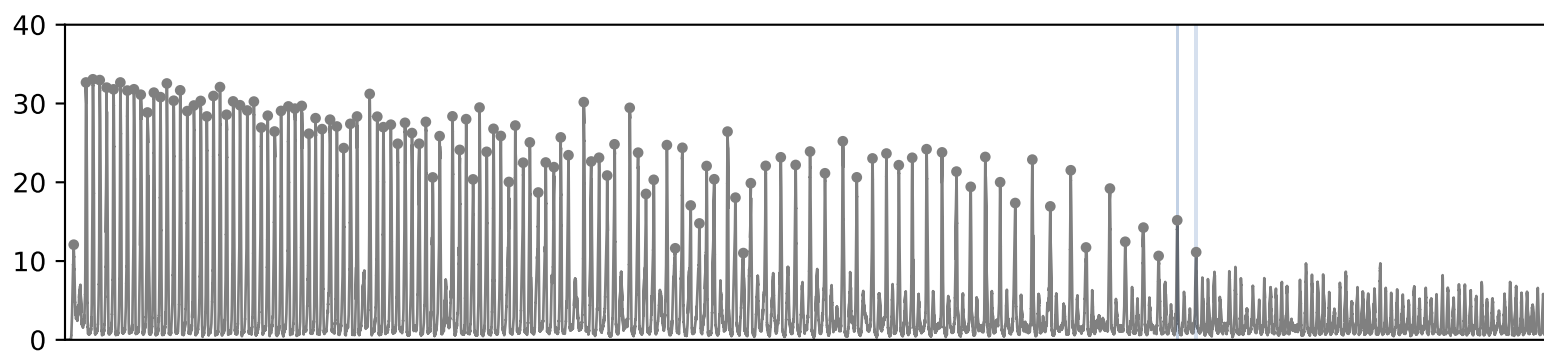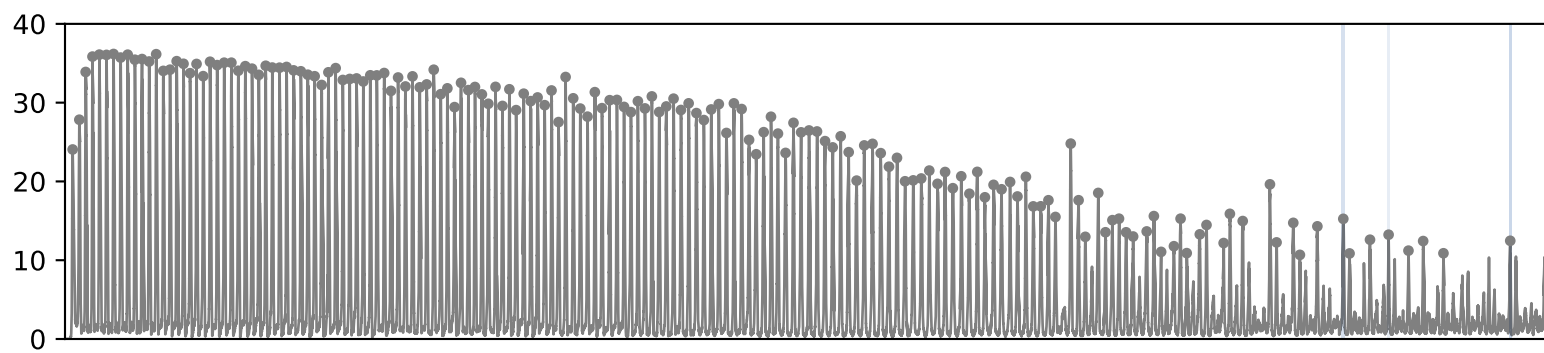

Supplement: Code files — Download Code files, ZIP file. [file eneuro-11-ENEURO.0284-23.2023-s001.zip › prebot-opioid-model-main/figure_notebooks/fig1/fig1_traces.pdf]

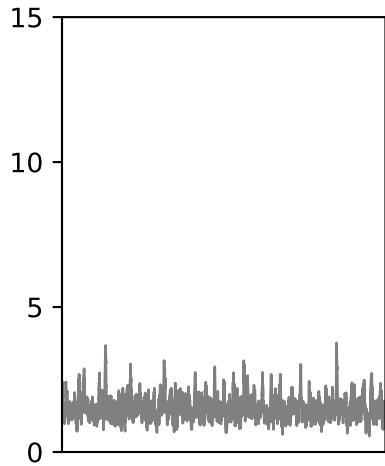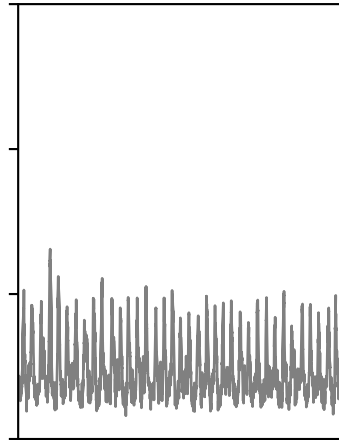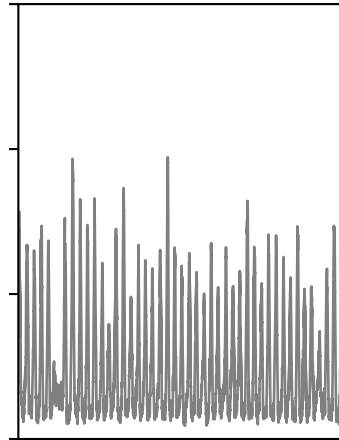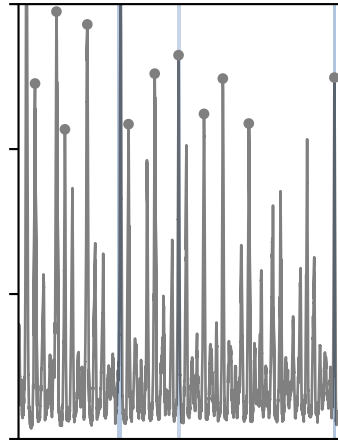

Supplement: Code files — Download Code files, ZIP file. [file eneuro-11-ENEURO.0284-23.2023-s001.zip › prebot-opioid-model-main/figure_notebooks/fig1/fig1_zoomed_in_traces.pdf]

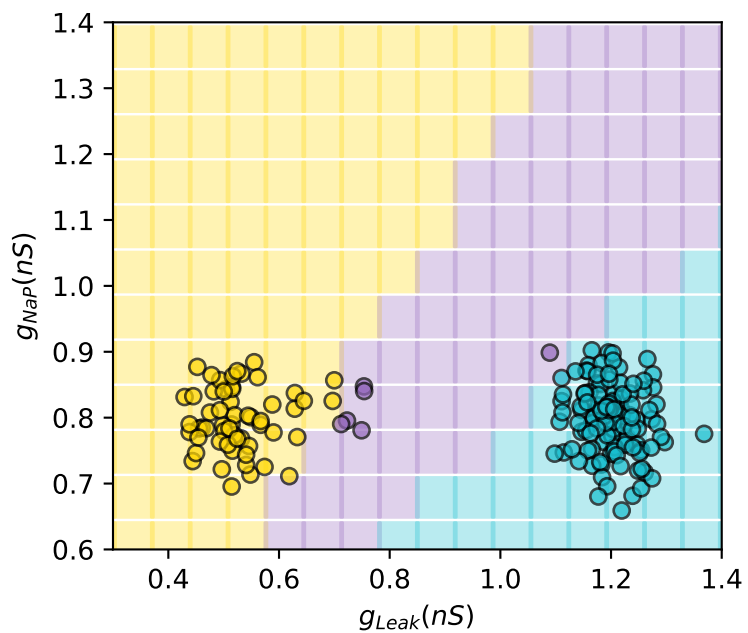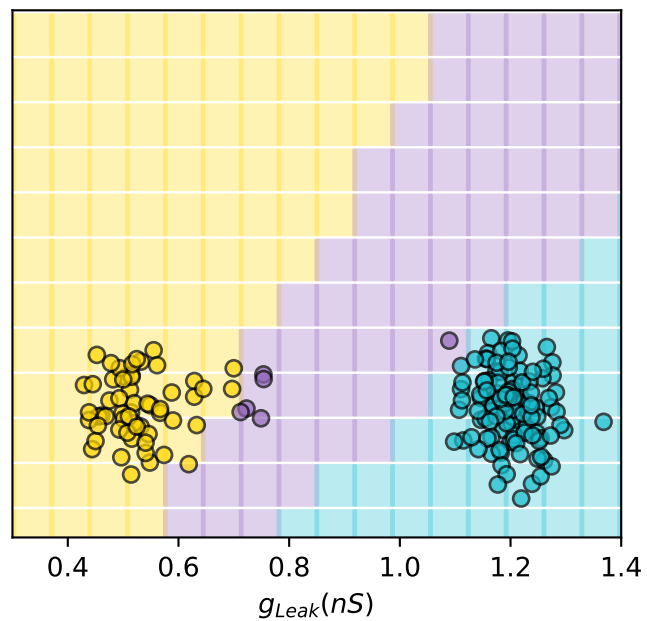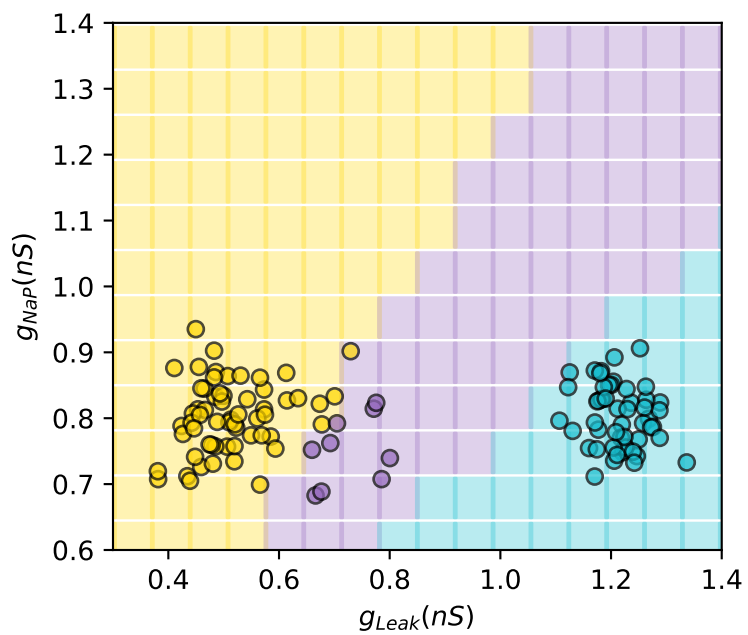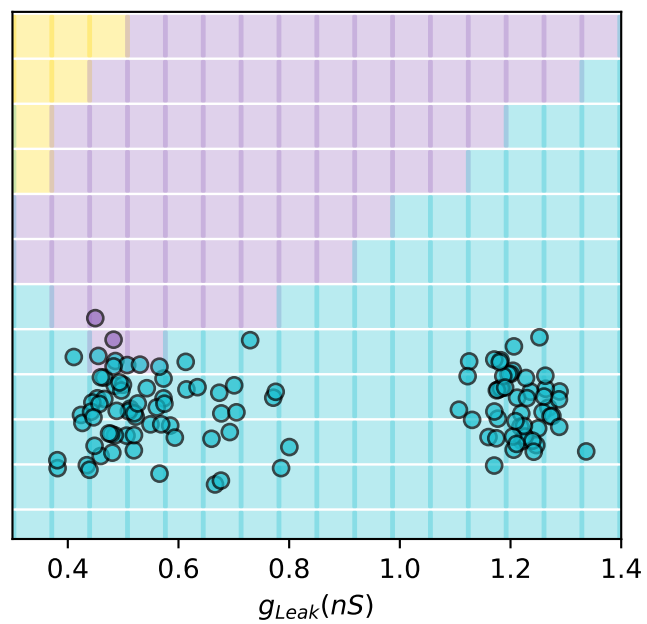

Supplement: Code files — Download Code files, ZIP file. [file eneuro-11-ENEURO.0284-23.2023-s001.zip › prebot-opioid-model-main/figure_notebooks/fig2/fig2_phase_diagrams_high_sensitivity.pdf]

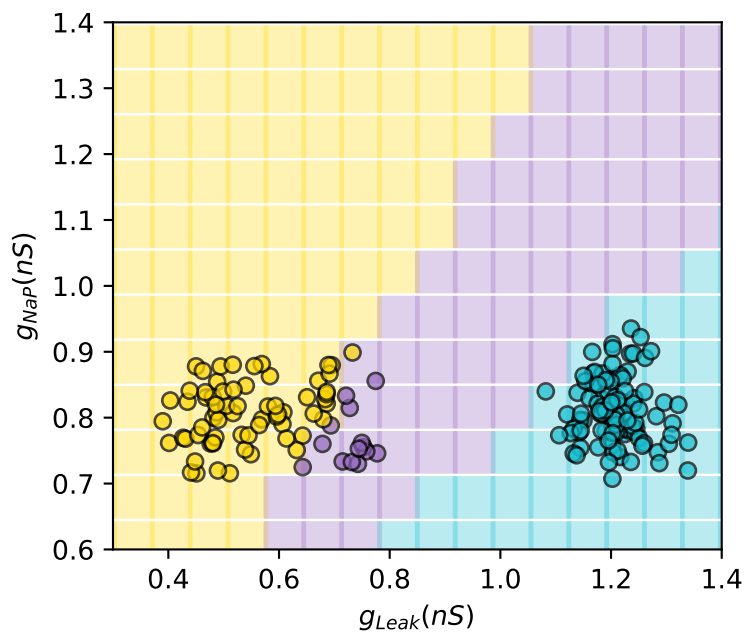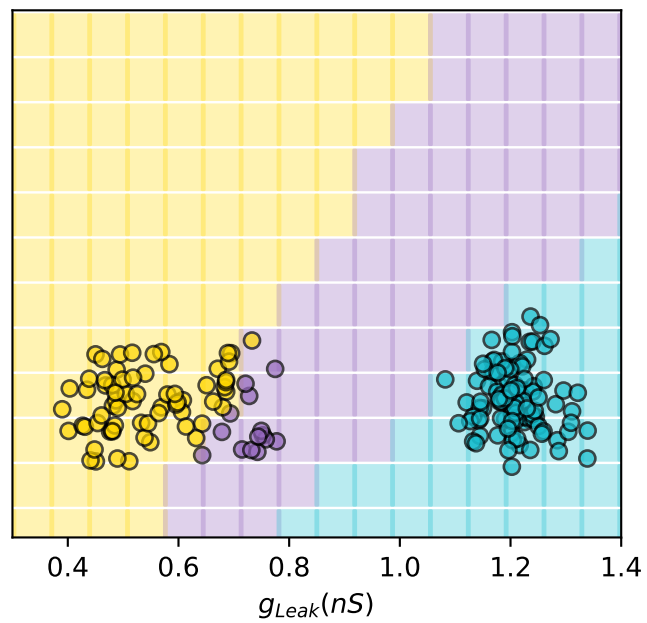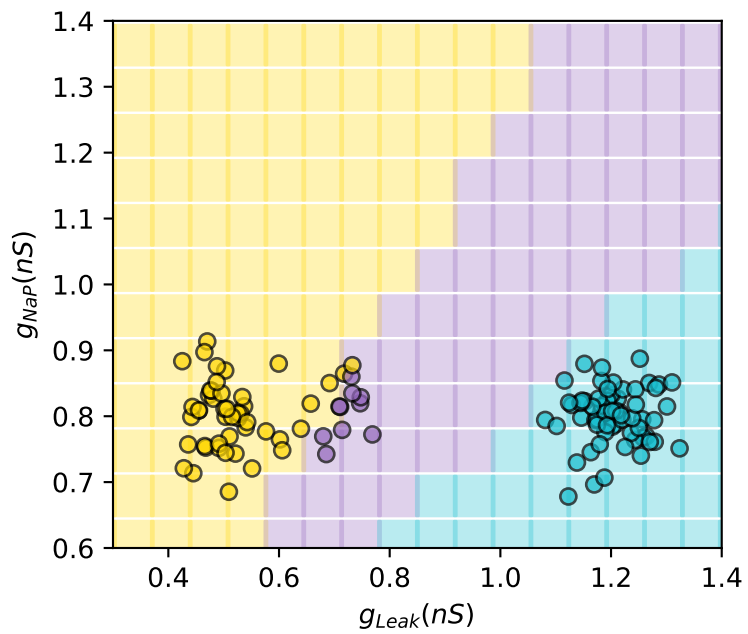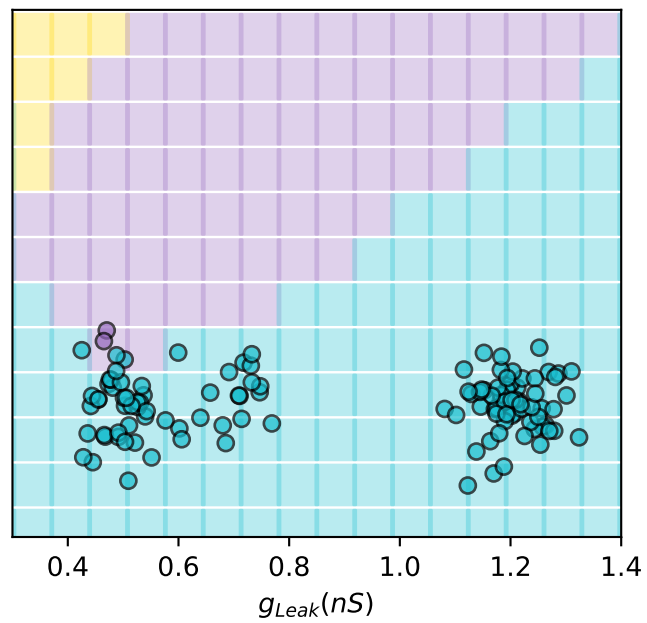

Supplement: Code files — Download Code files, ZIP file. [file eneuro-11-ENEURO.0284-23.2023-s001.zip › prebot-opioid-model-main/figure_notebooks/fig2/fig2_phase_diagrams_low_sensitivity.pdf]

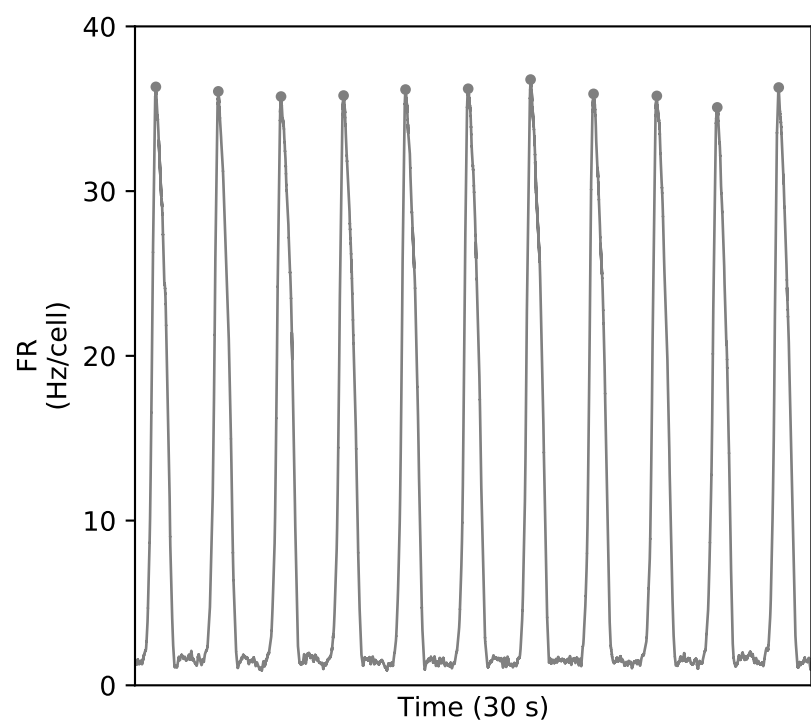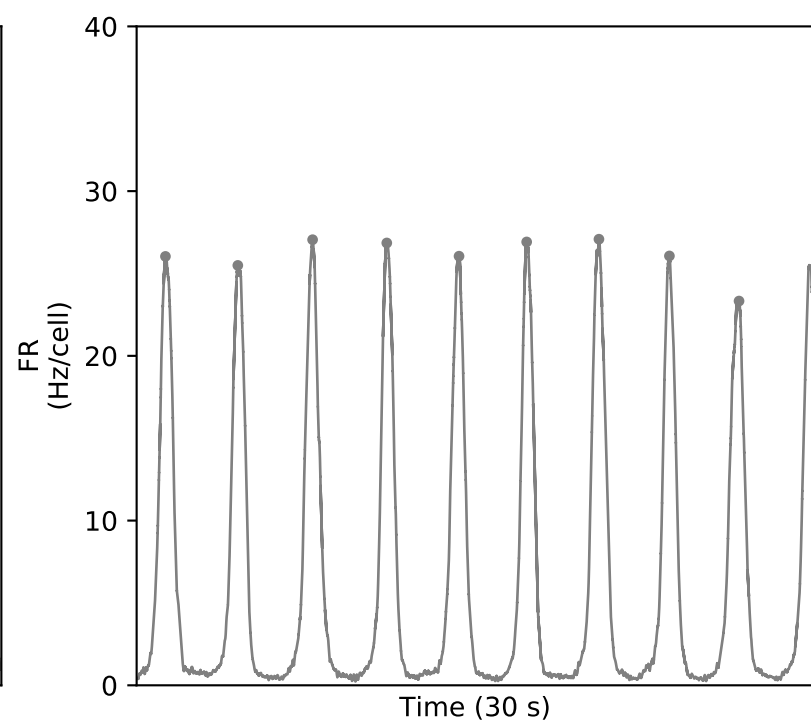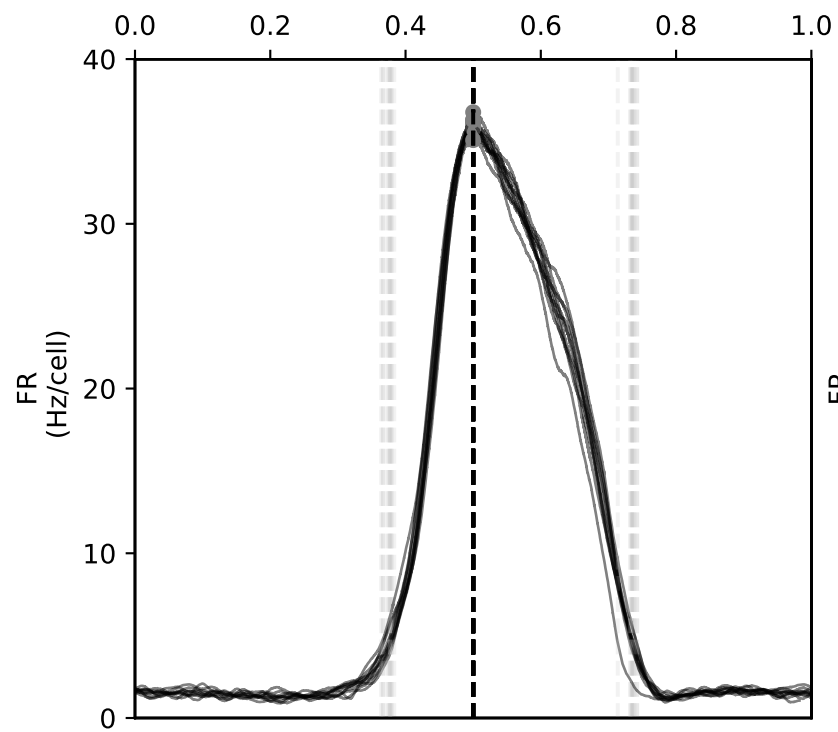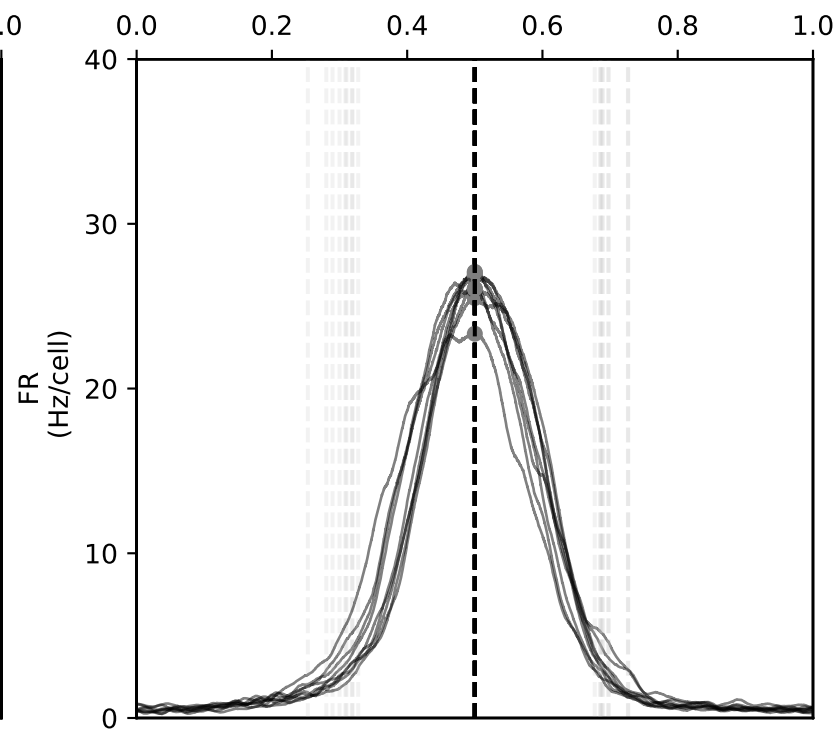

Supplement: Code files — Download Code files, ZIP file. [file eneuro-11-ENEURO.0284-23.2023-s001.zip › prebot-opioid-model-main/figure_notebooks/fig2/fig2_poprate_high_sensitivity.pdf]

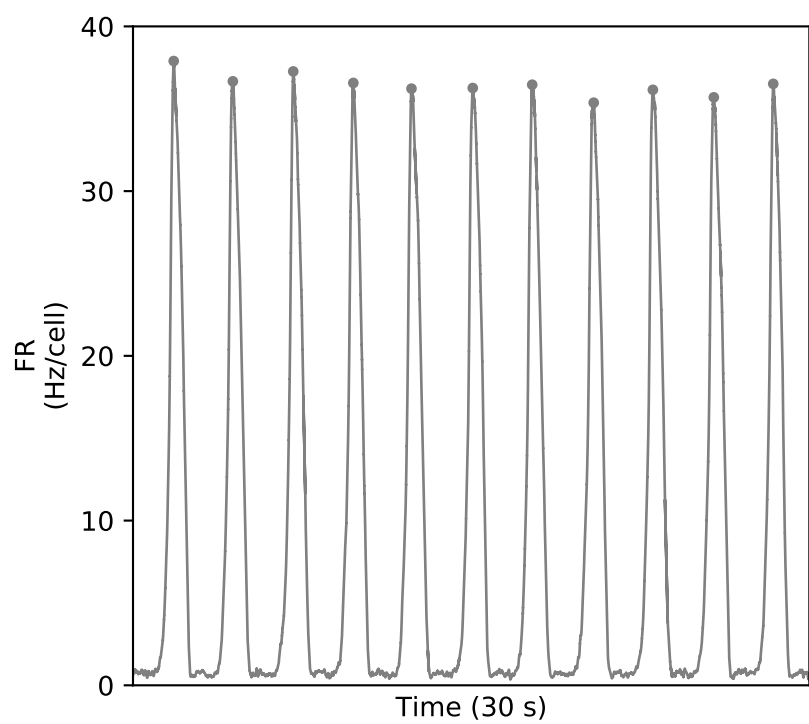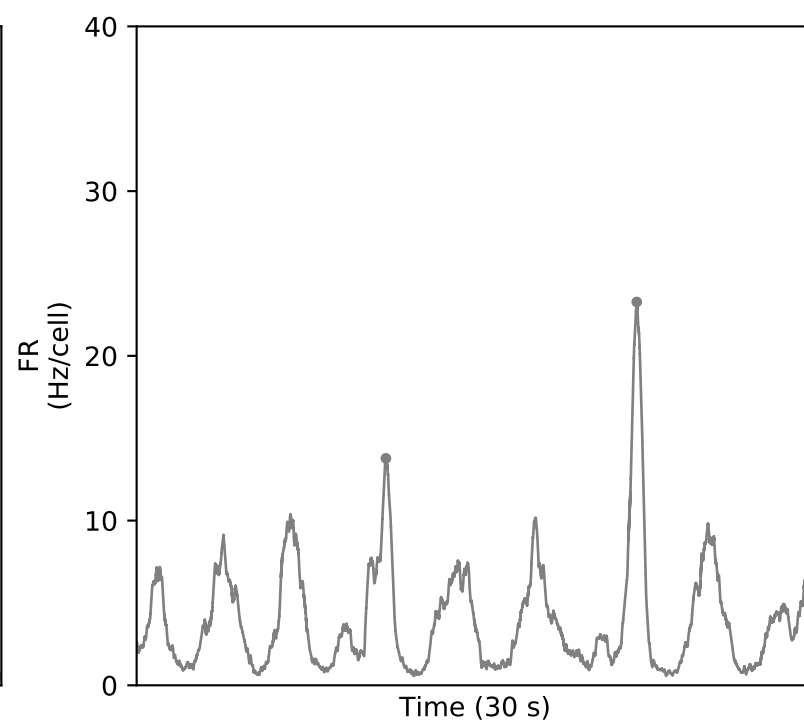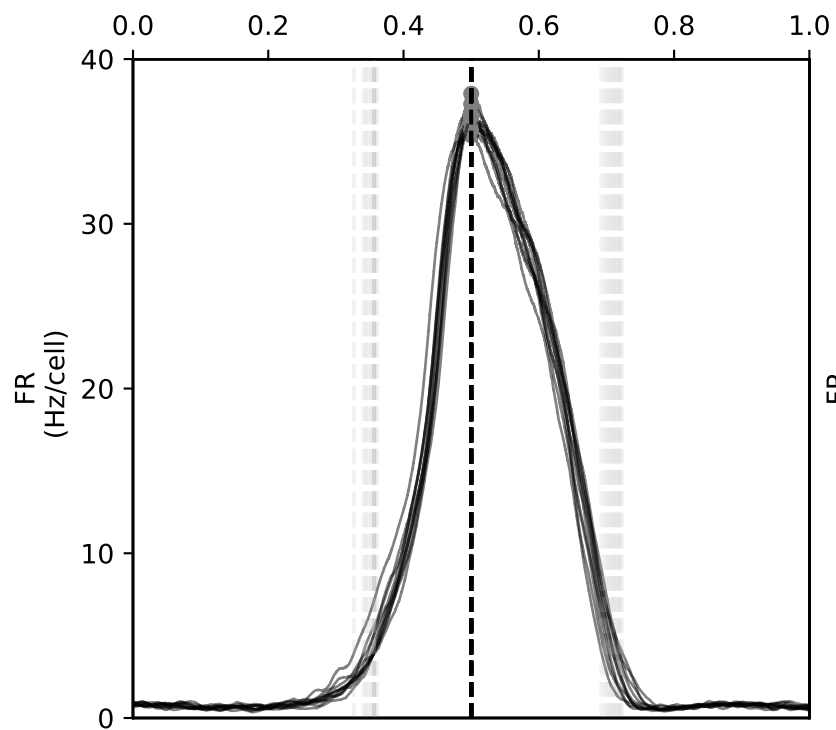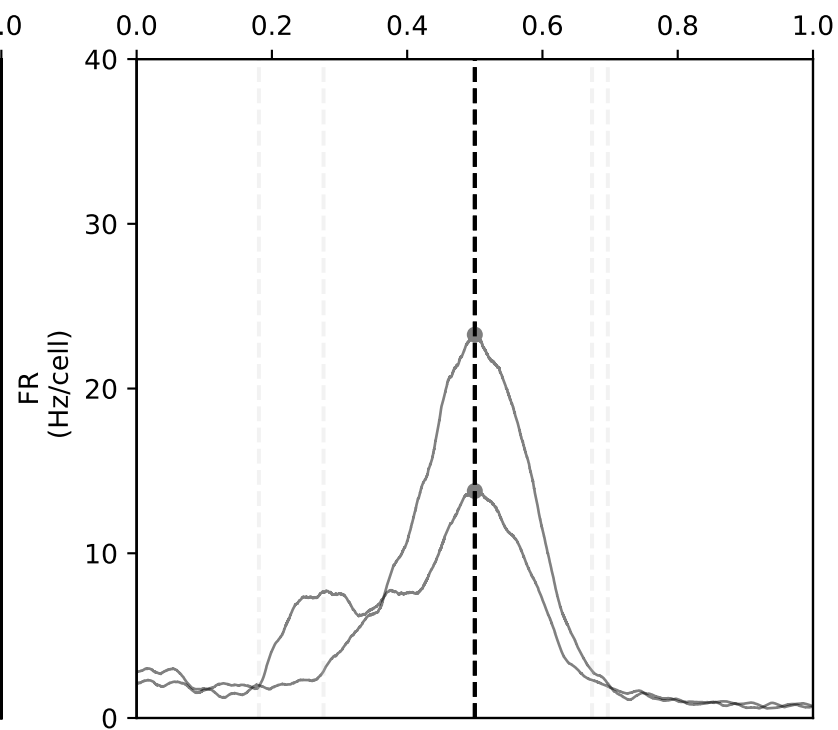

Supplement: Code files — Download Code files, ZIP file. [file eneuro-11-ENEURO.0284-23.2023-s001.zip › prebot-opioid-model-main/figure_notebooks/fig2/fig2_poprate_low_sensitivity.pdf]

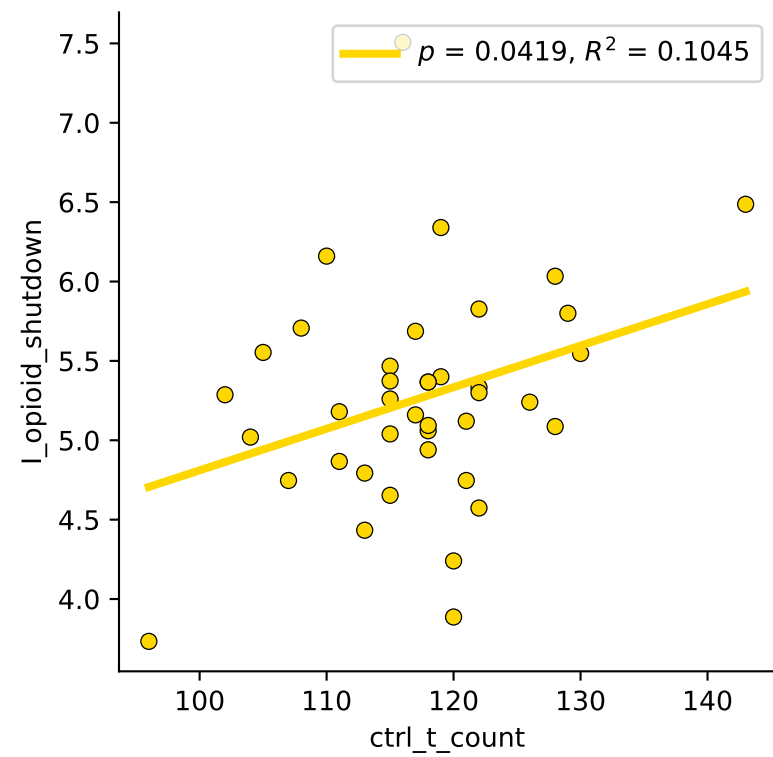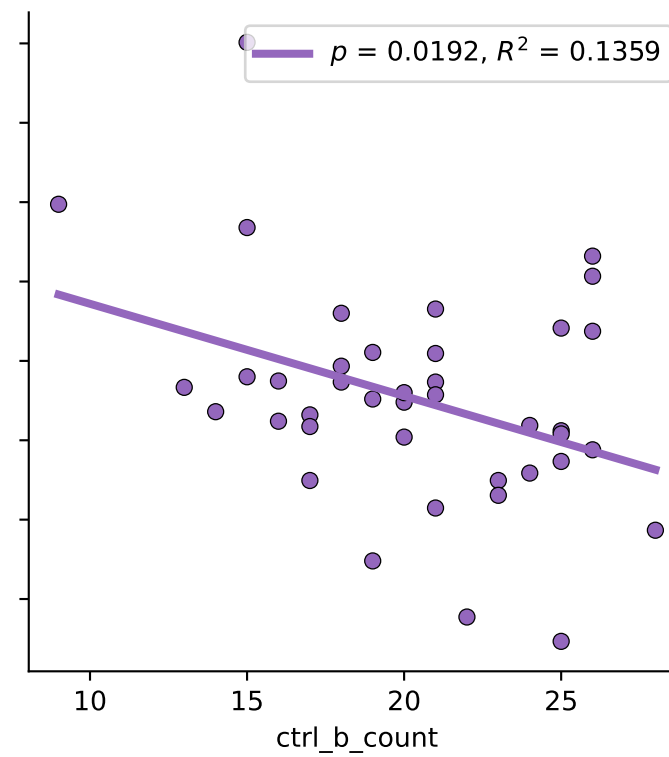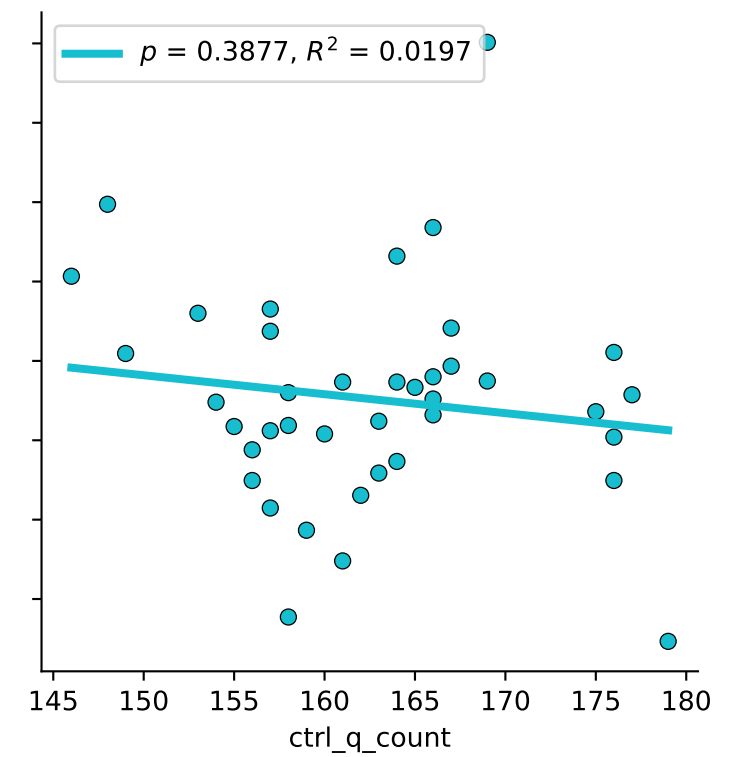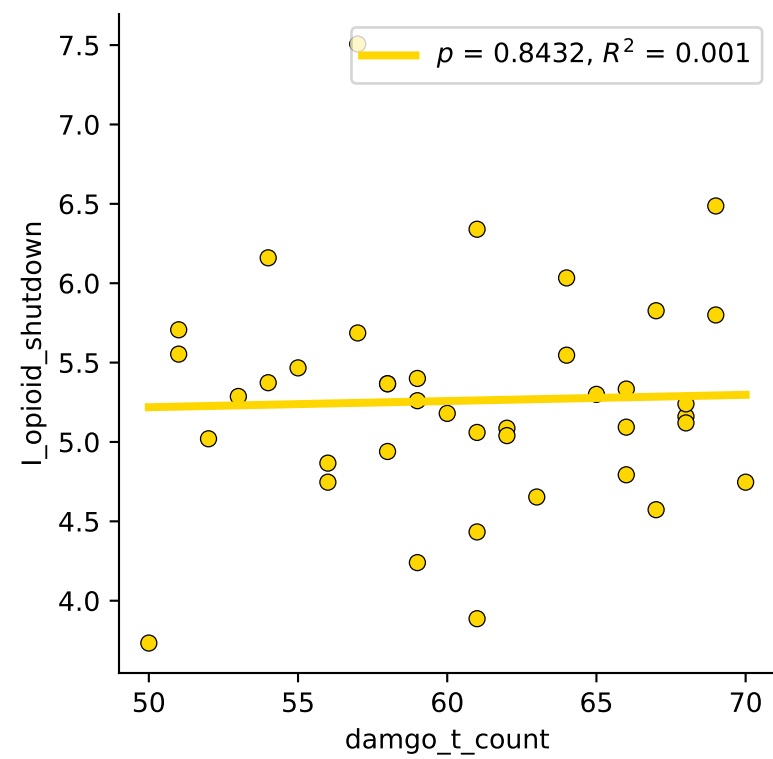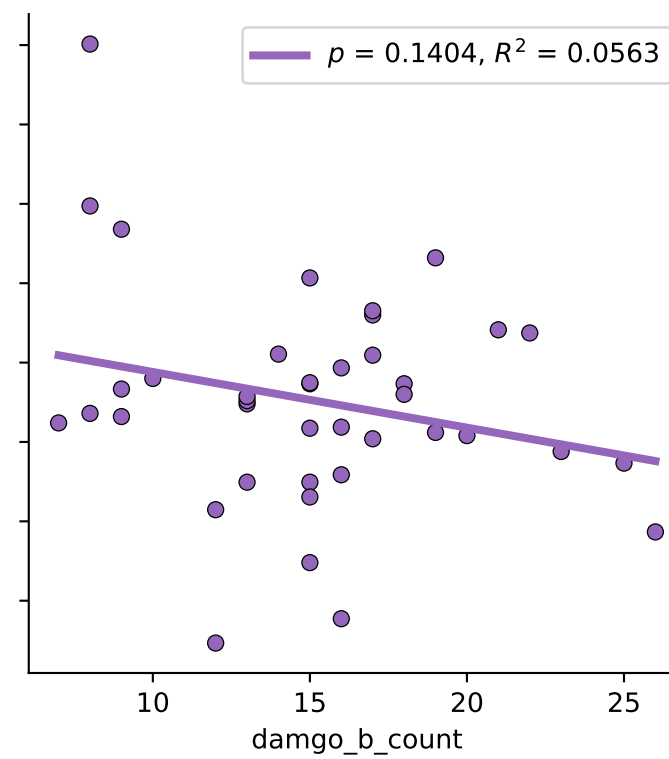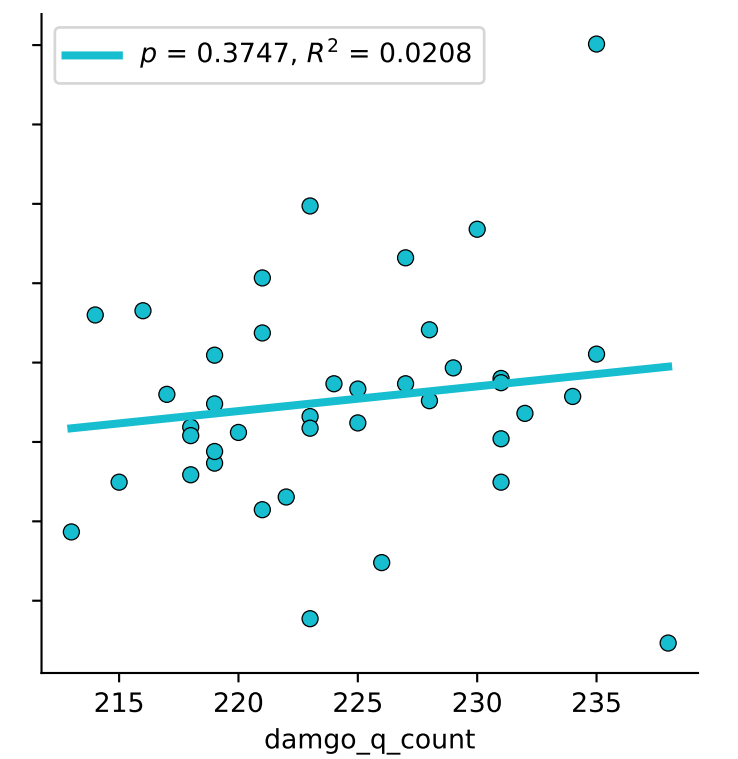

Supplement: Code files — Download Code files, ZIP file. [file eneuro-11-ENEURO.0284-23.2023-s001.zip › prebot-opioid-model-main/figure_notebooks/fig2/fig2_tbq_vs_shutdown.pdf]

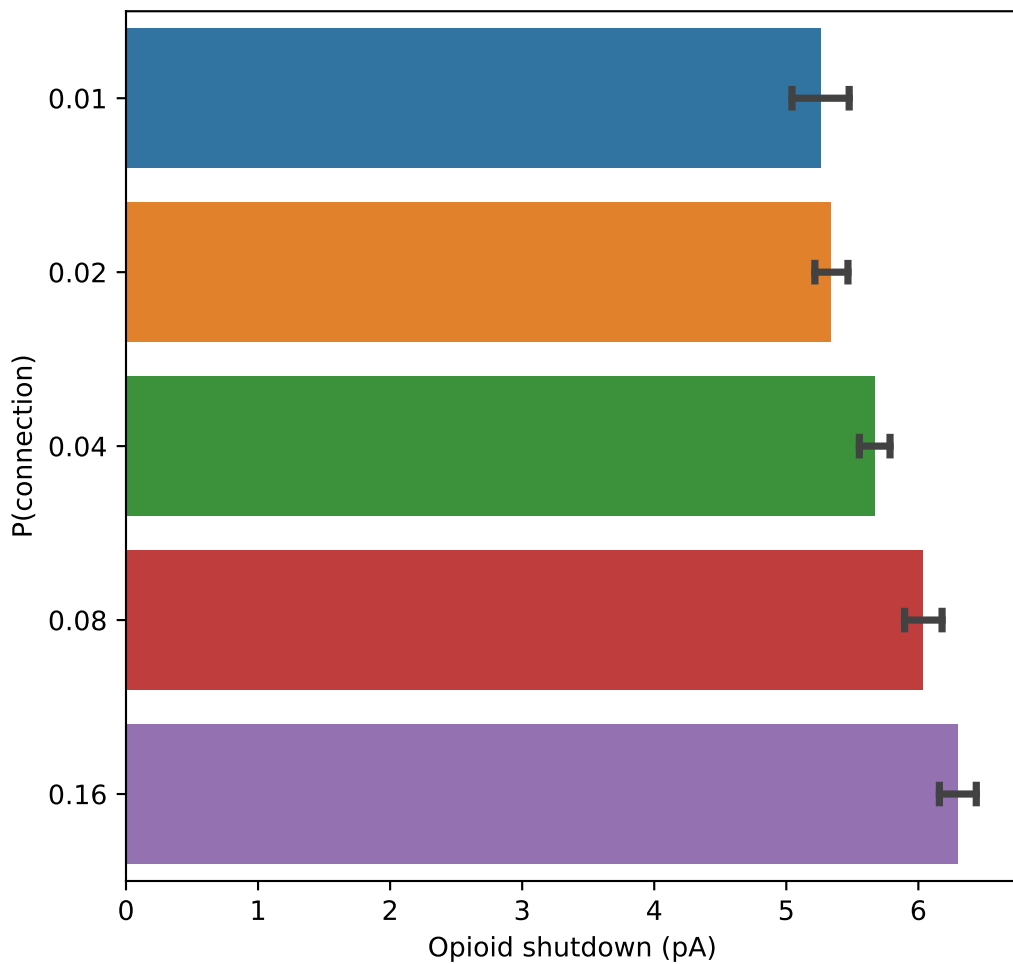

Supplement: Code files — Download Code files, ZIP file. [file eneuro-11-ENEURO.0284-23.2023-s001.zip › prebot-opioid-model-main/figure_notebooks/fig3/fig3_barplot.pdf]

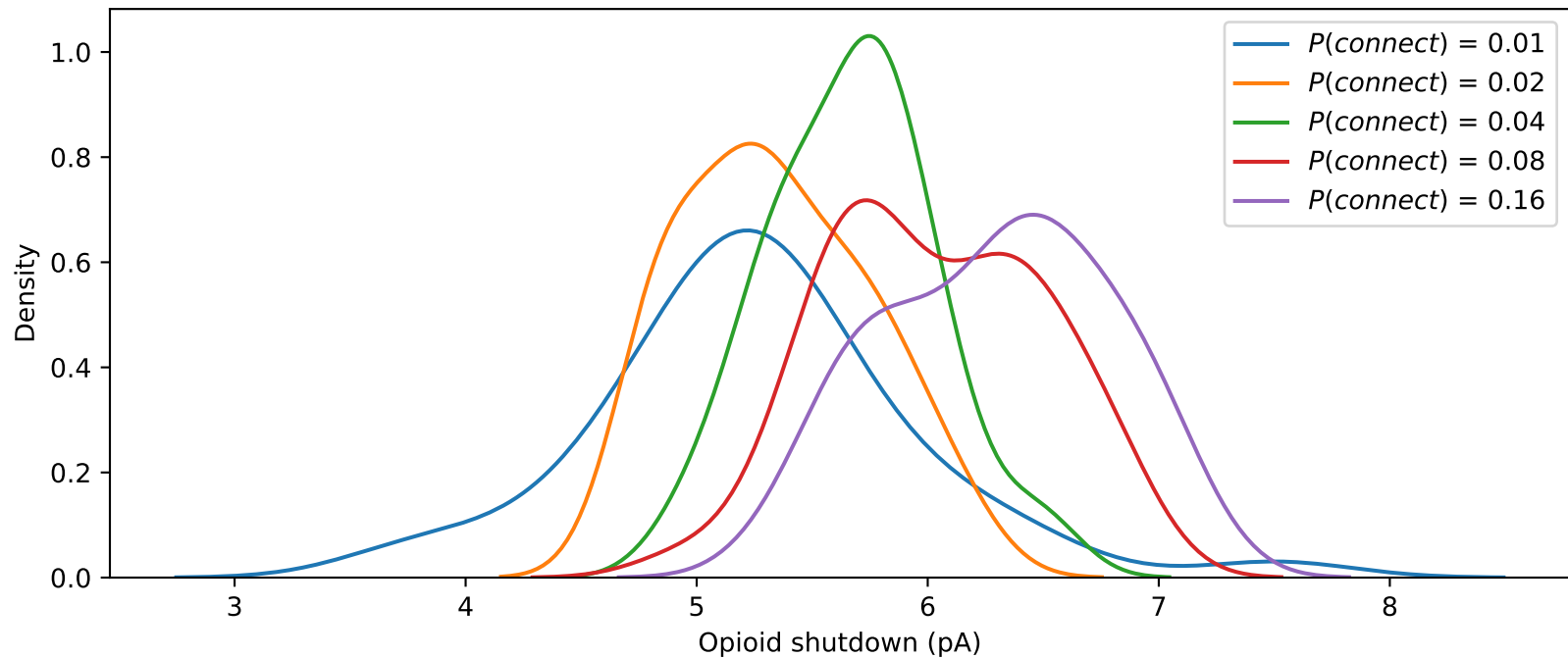

Supplement: Code files — Download Code files, ZIP file. [file eneuro-11-ENEURO.0284-23.2023-s001.zip › prebot-opioid-model-main/figure_notebooks/fig3/fig3_shutdown_distr.pdf]

$P(\text{connect}) = 0.01$

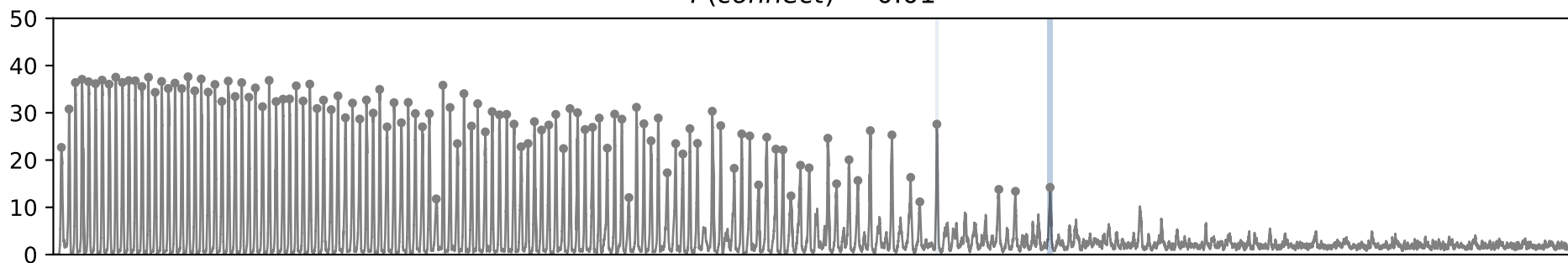

$P(\text{connect}) = 0.02$

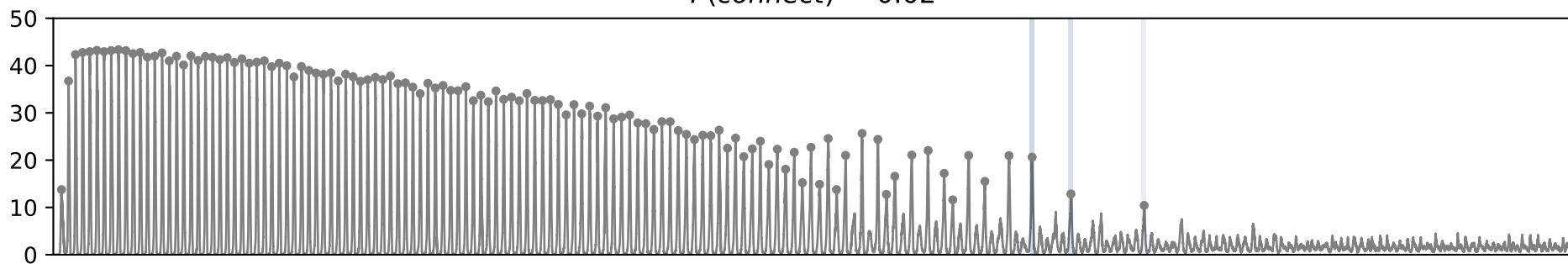

$P(\text{connect}) = 0.04$

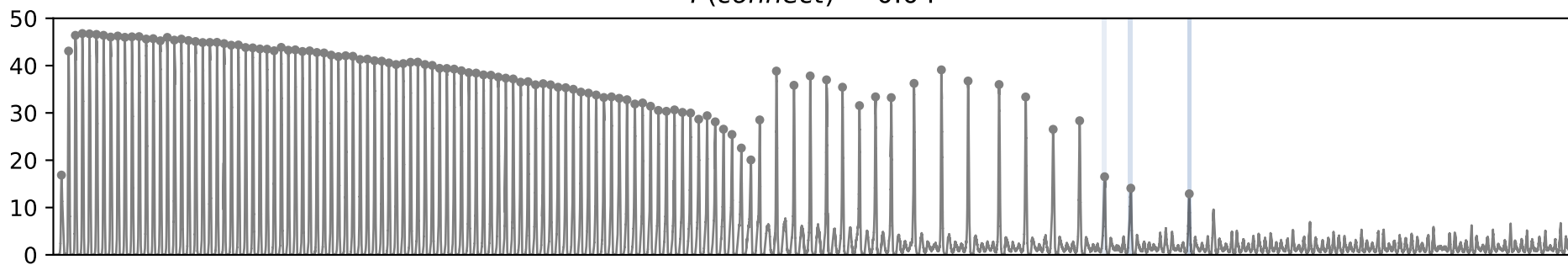

$P(\text{connect}) = 0.08$

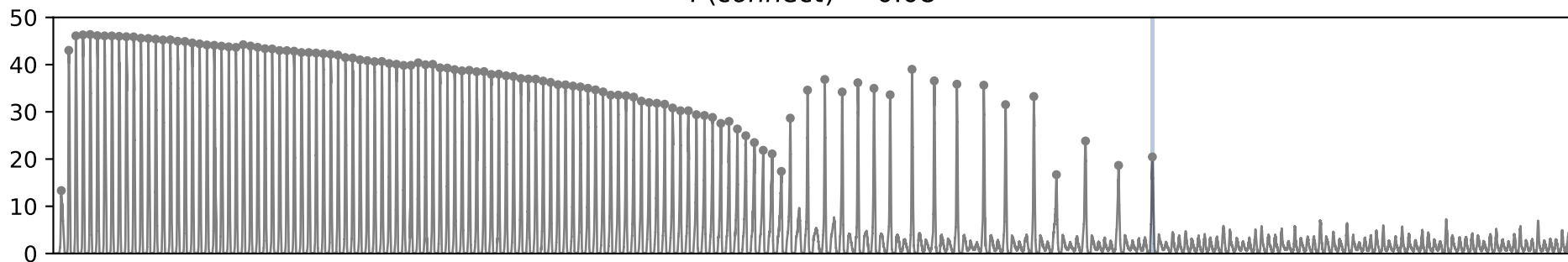

$P(\text{connect}) = 0.16$

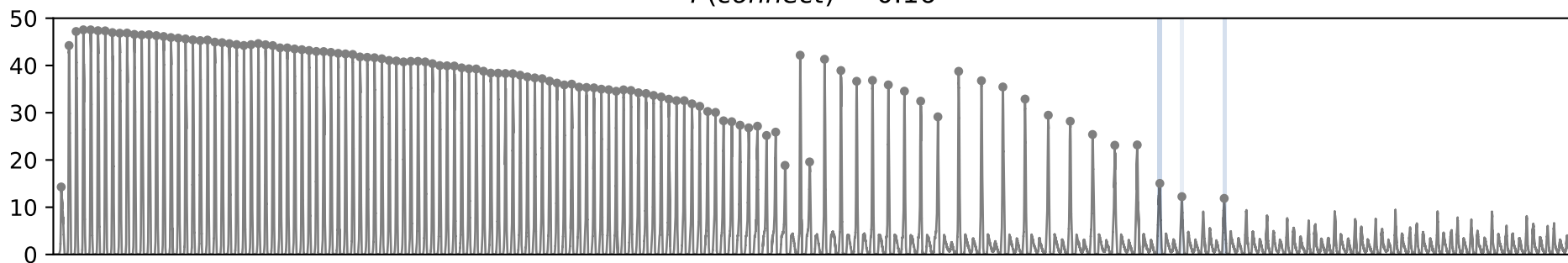

Time (10 minutes)

Supplement: Code files — Download Code files, ZIP file. [file eneuro-11-ENEURO.0284-23.2023-s001.zip › prebot-opioid-model-main/figure_notebooks/fig3/fig3_traces.pdf]

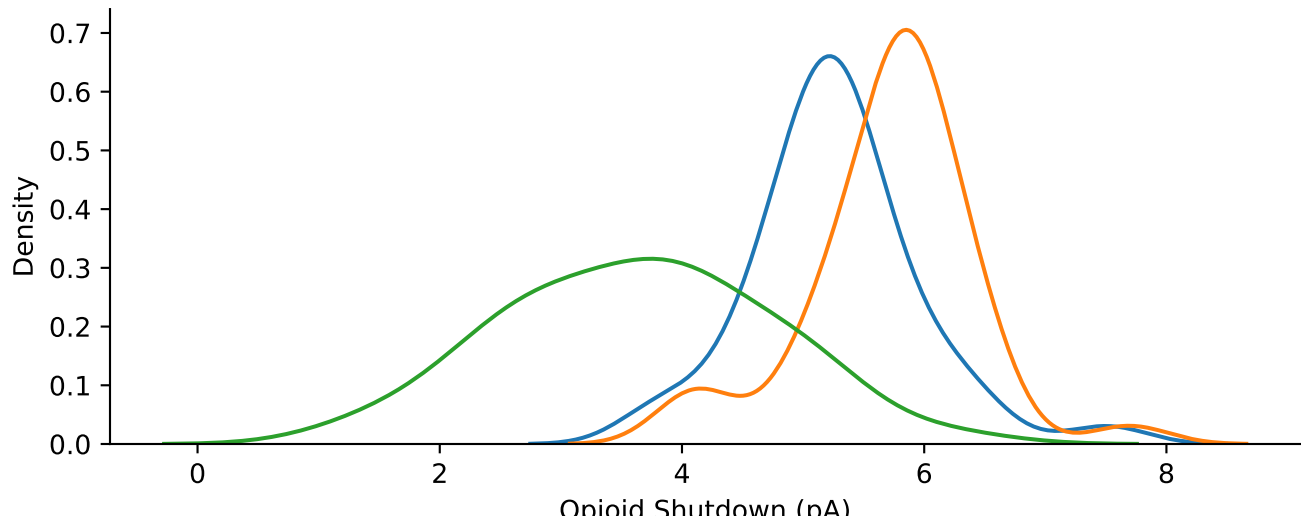

Supplement: Code files — Download Code files, ZIP file. [file eneuro-11-ENEURO.0284-23.2023-s001.zip › prebot-opioid-model-main/figure_notebooks/fig5/fig5_distr.pdf]

high  $G_L$

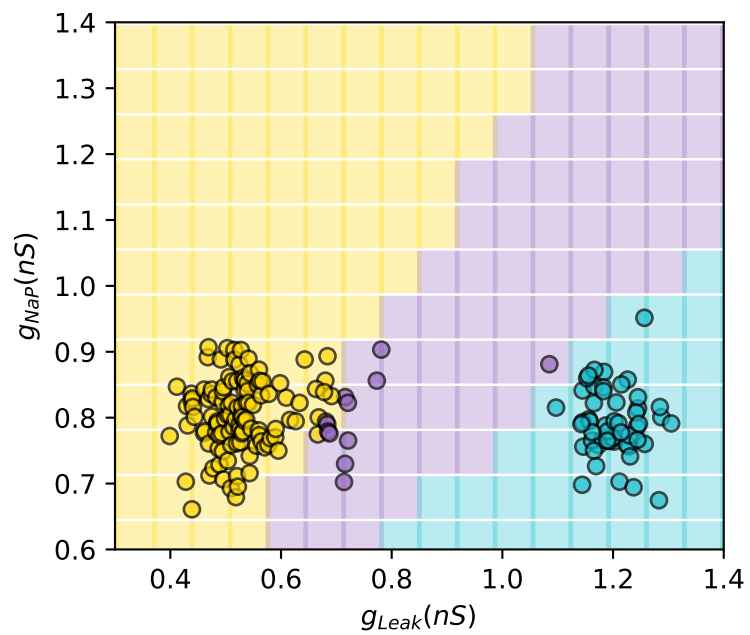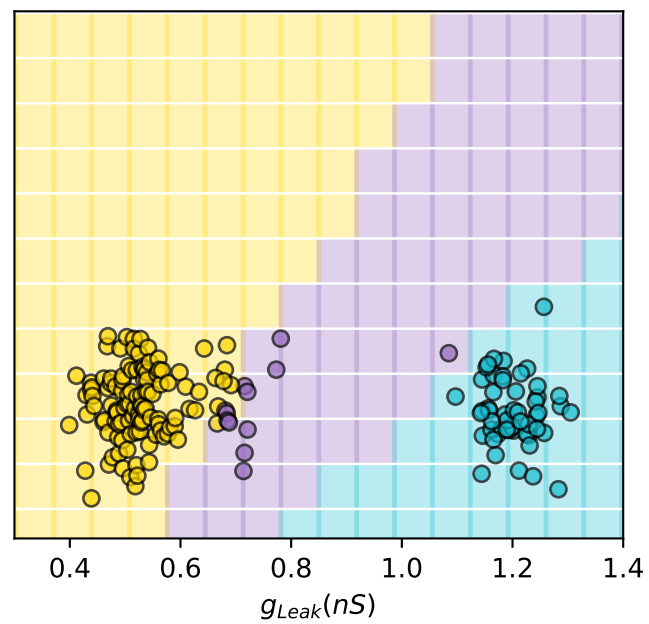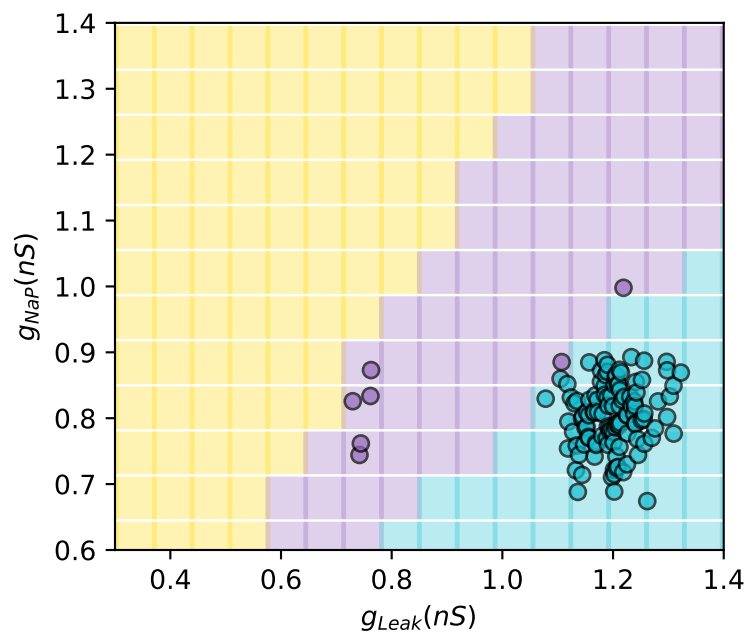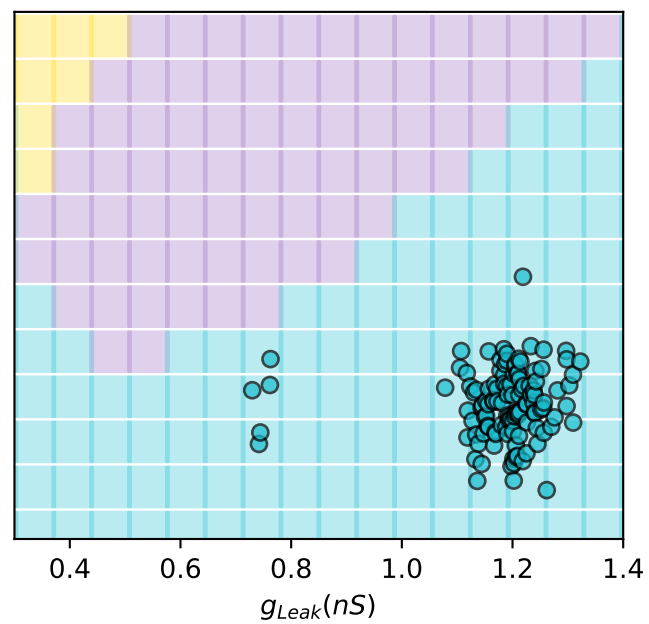

Supplement: Code files — Download Code files, ZIP file. [file eneuro-11-ENEURO.0284-23.2023-s001.zip › prebot-opioid-model-main/figure_notebooks/fig5/fig5_phase_diagrams_high_gleak.pdf]

low  $G_L$

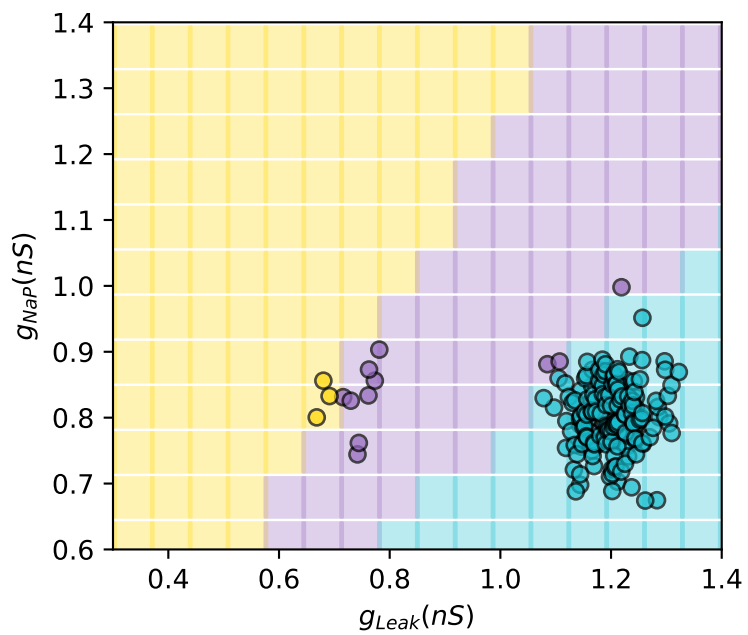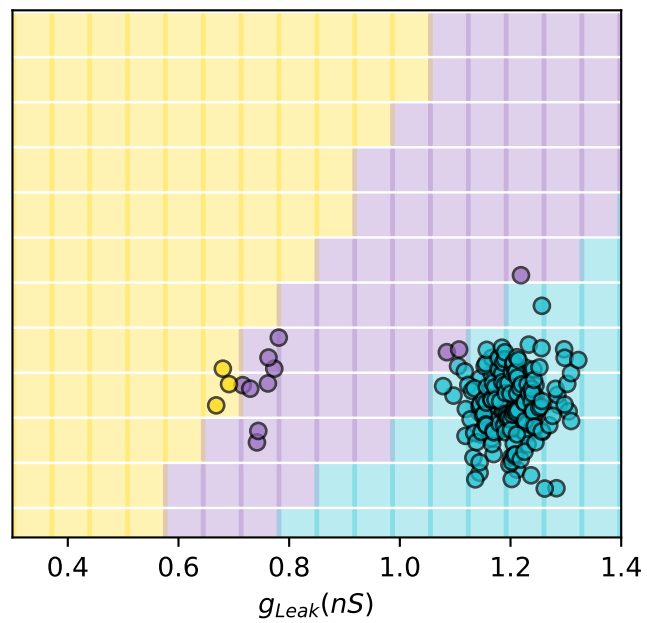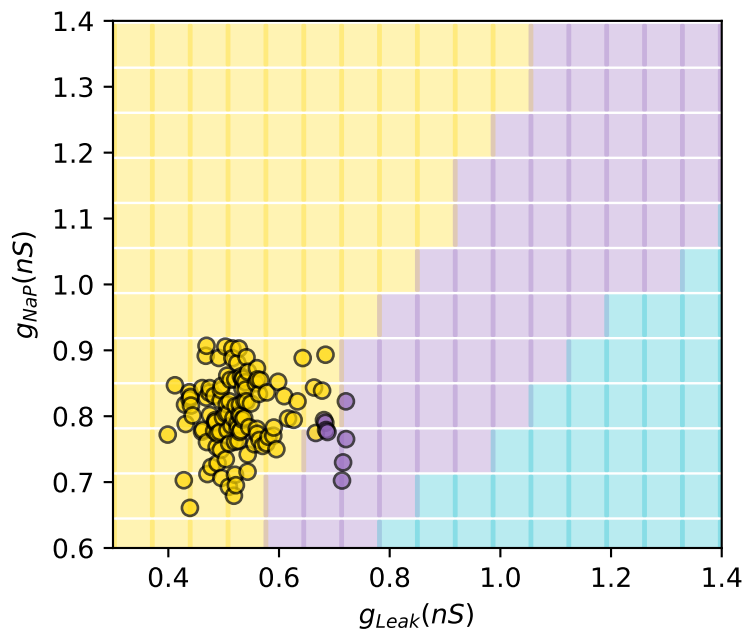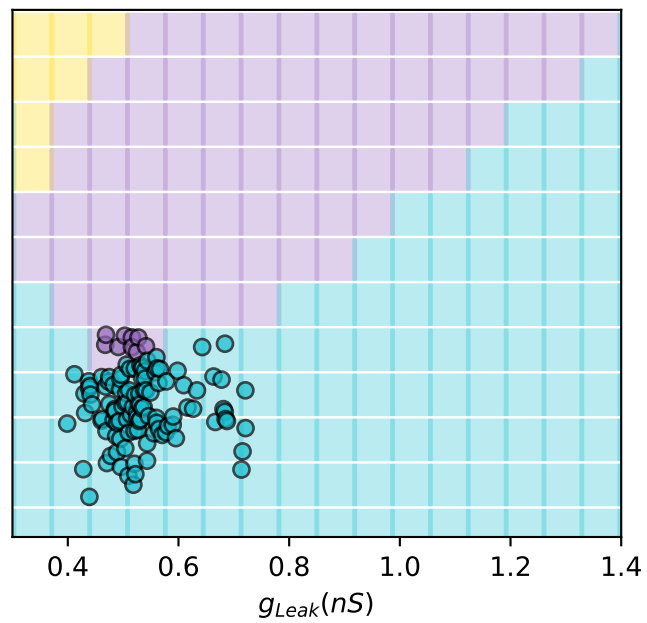

Supplement: Code files — Download Code files, ZIP file. [file eneuro-11-ENEURO.0284-23.2023-s001.zip › prebot-opioid-model-main/figure_notebooks/fig5/fig5_phase_diagrams_low_gleak.pdf]

random  $G_L$ 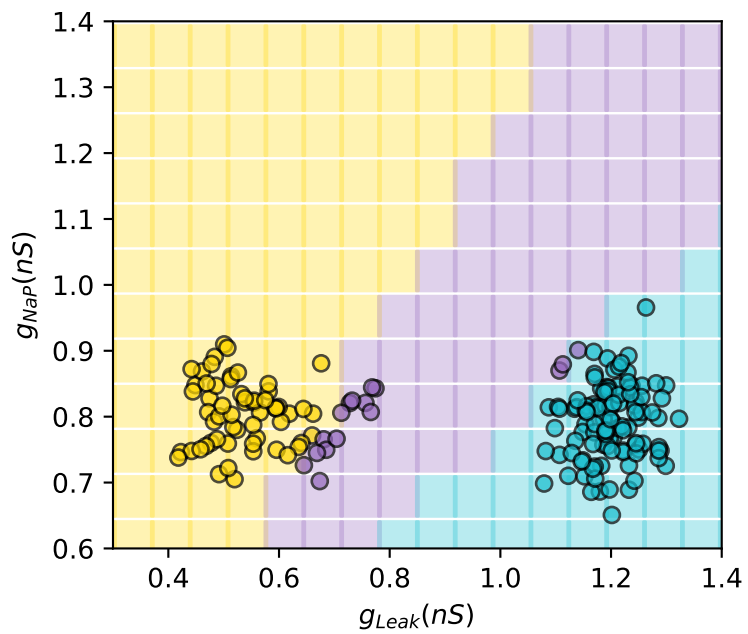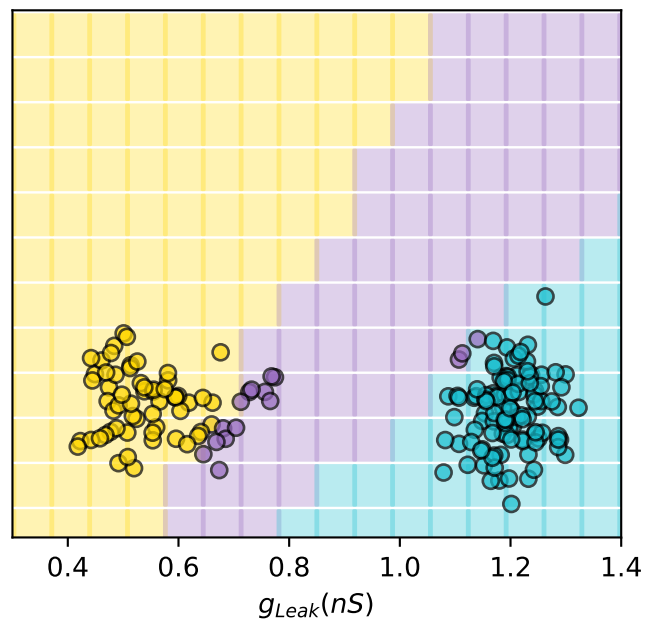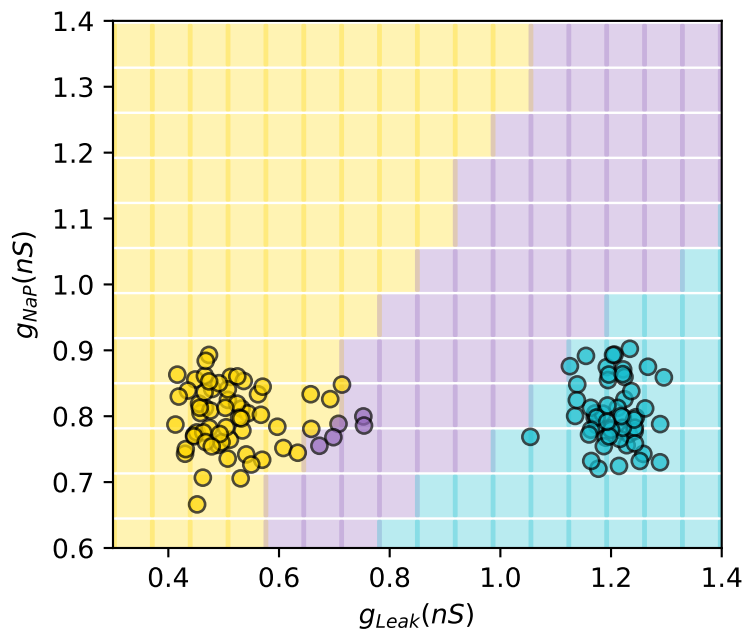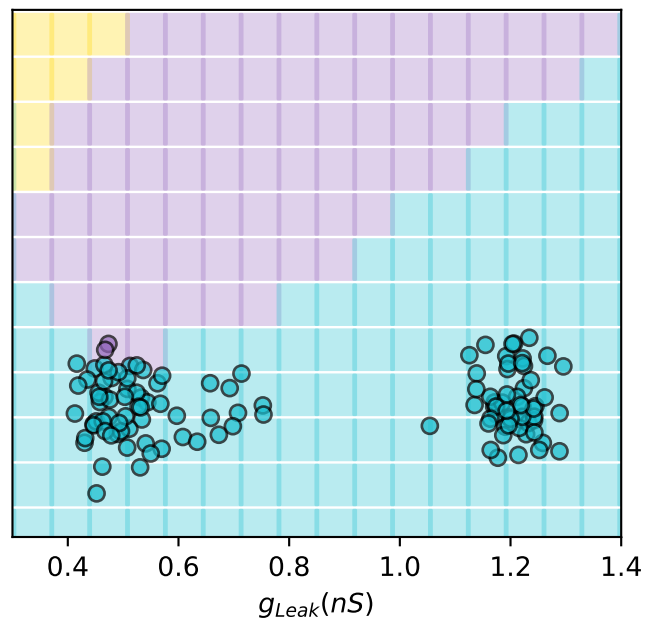

Supplement: Code files — Download Code files, ZIP file. [file eneuro-11-ENEURO.0284-23.2023-s001.zip › prebot-opioid-model-main/figure_notebooks/fig5/fig5_phase_diagrams_random_gleak.pdf]

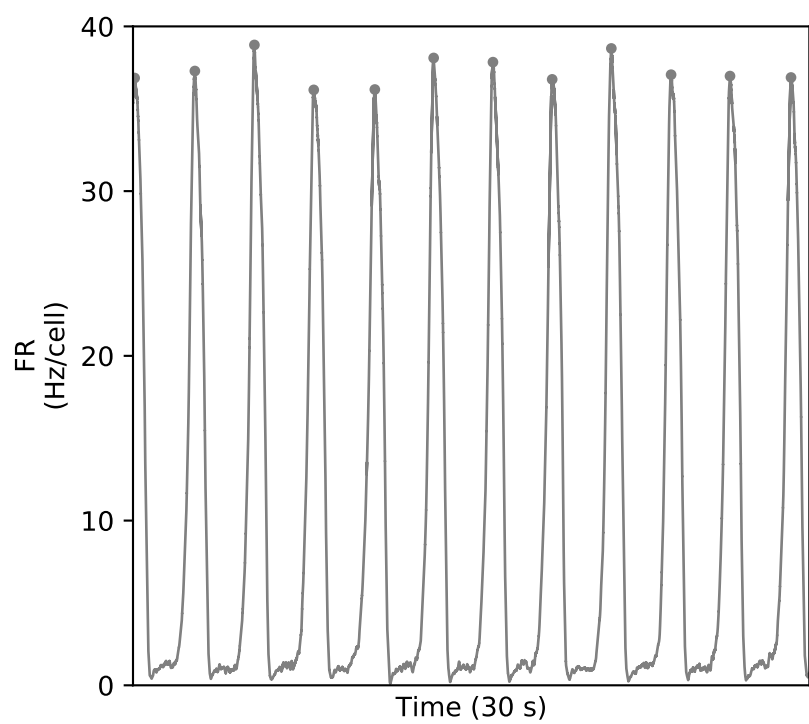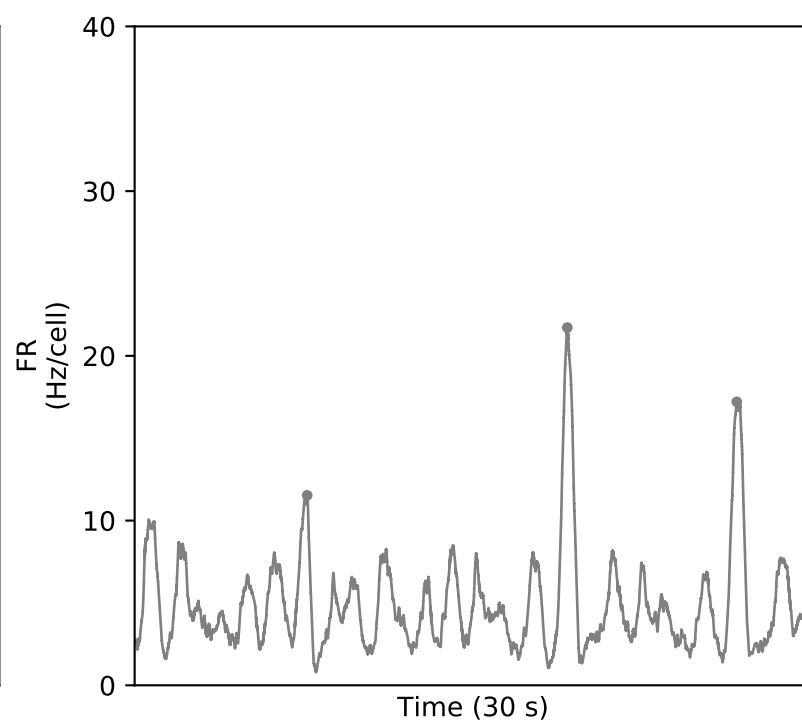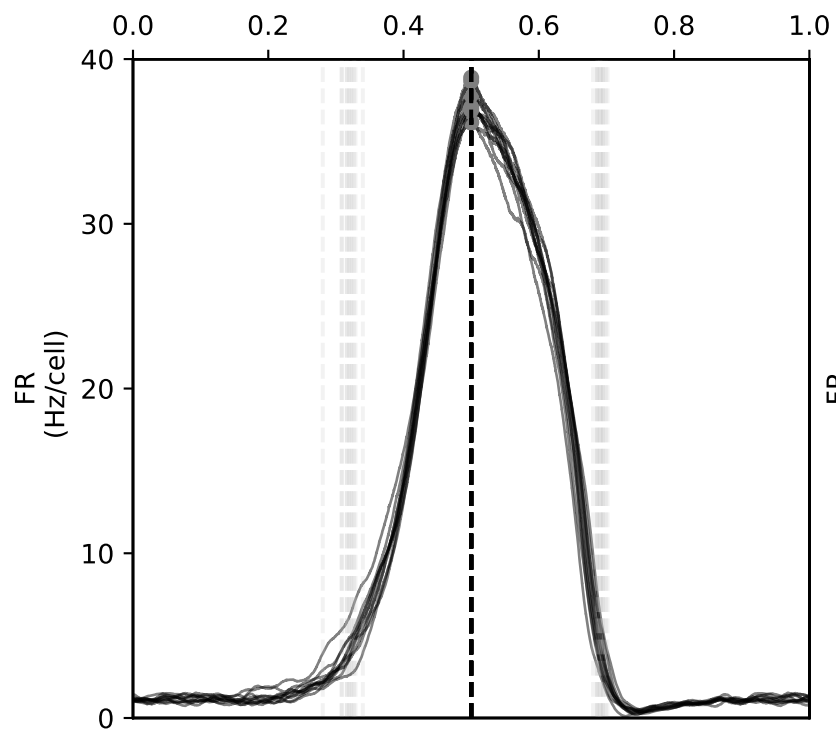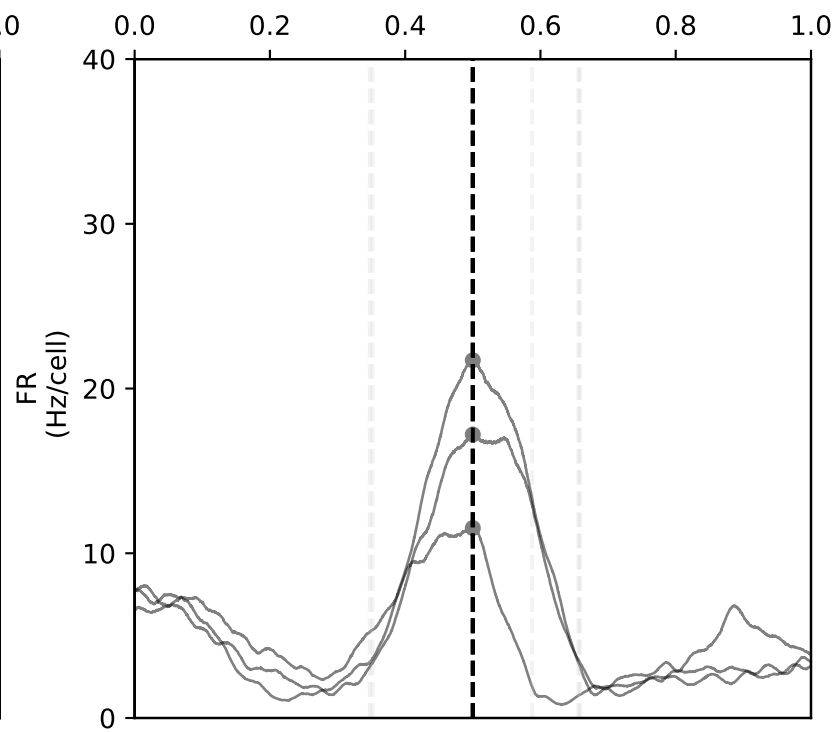

Supplement: Code files — Download Code files, ZIP file. [file eneuro-11-ENEURO.0284-23.2023-s001.zip › prebot-opioid-model-main/figure_notebooks/fig5/fig5_poprate_high_gleak.pdf]

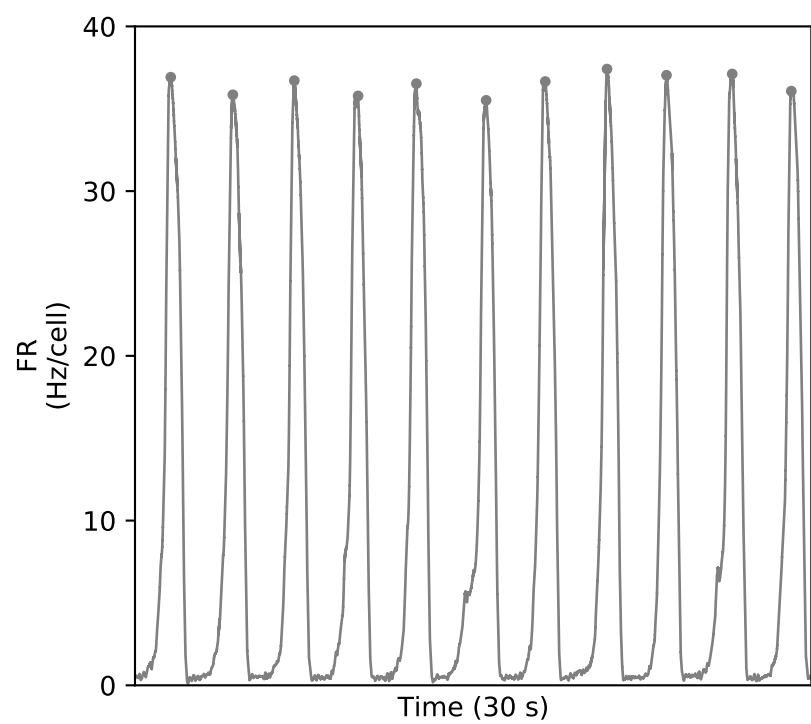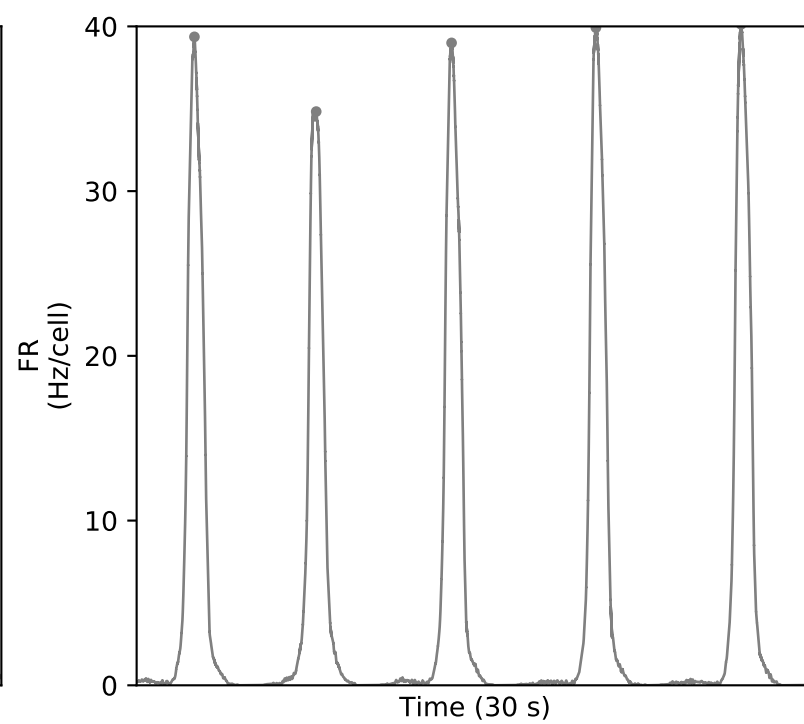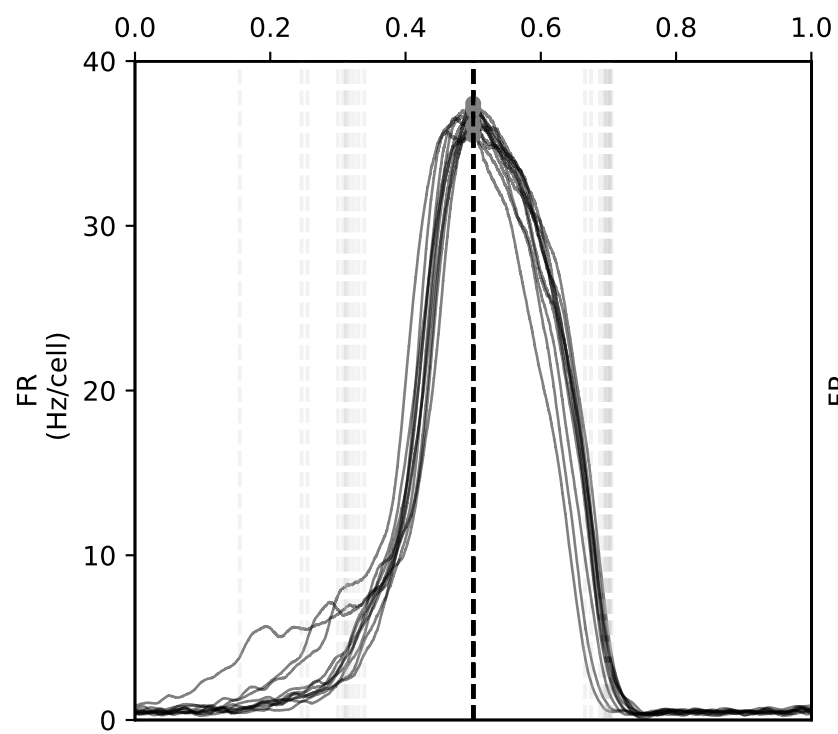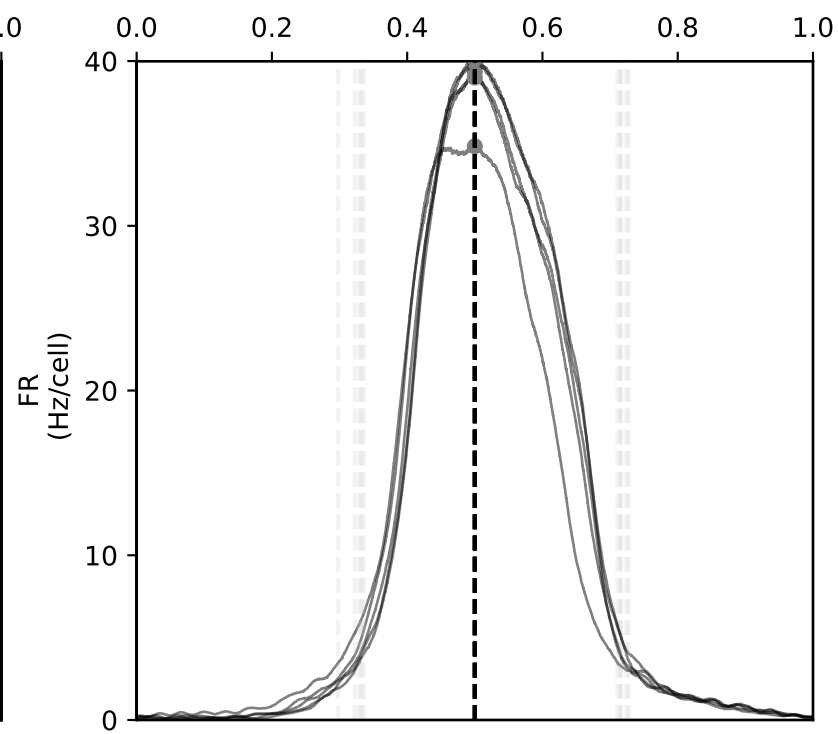

Supplement: Code files — Download Code files, ZIP file. [file eneuro-11-ENEURO.0284-23.2023-s001.zip › prebot-opioid-model-main/figure_notebooks/fig5/fig5_poprate_low_gleak.pdf]

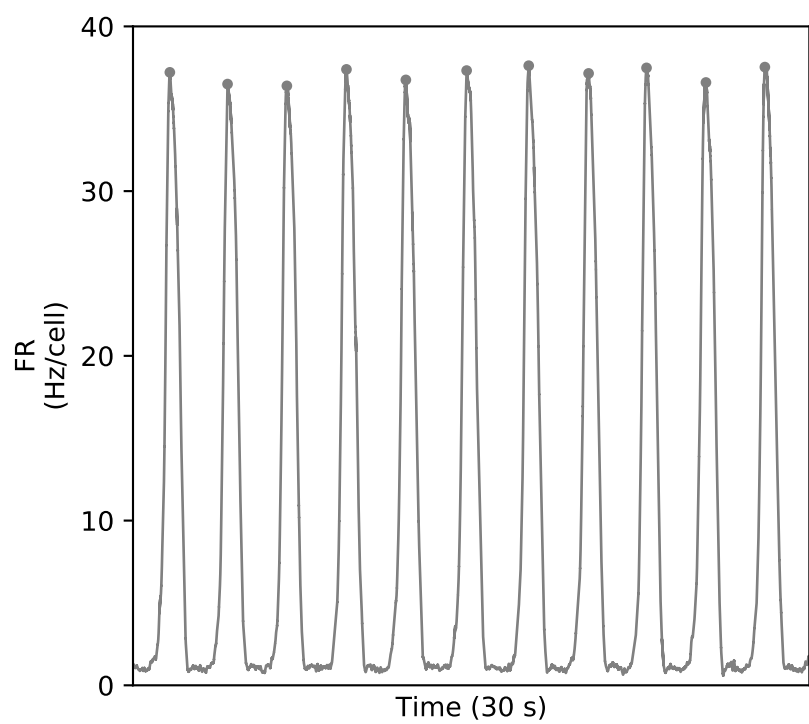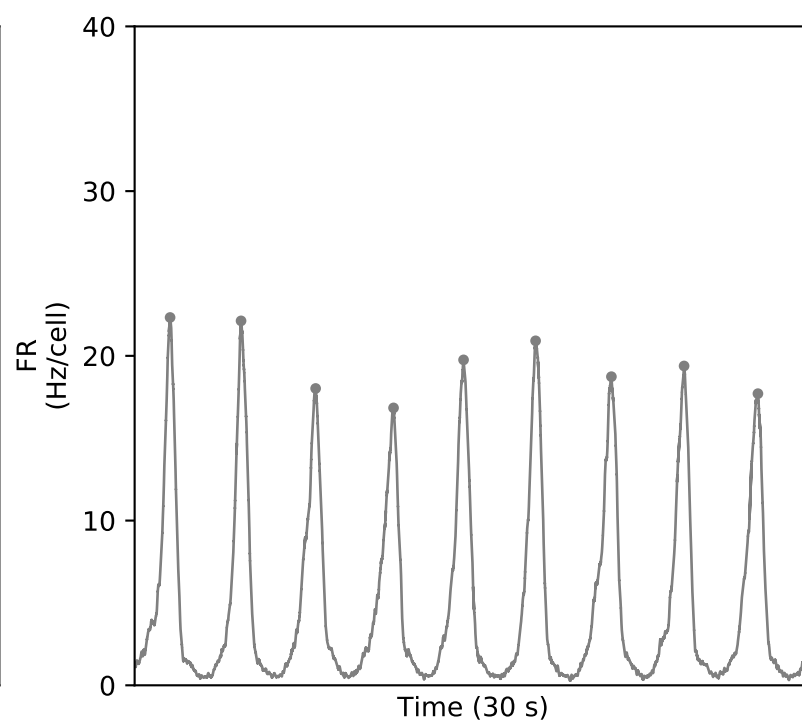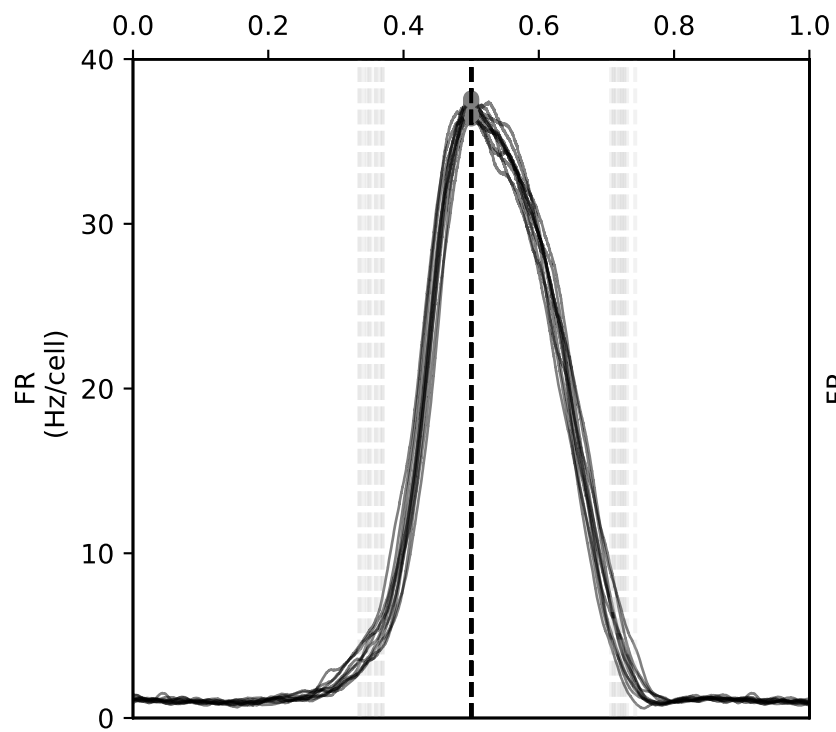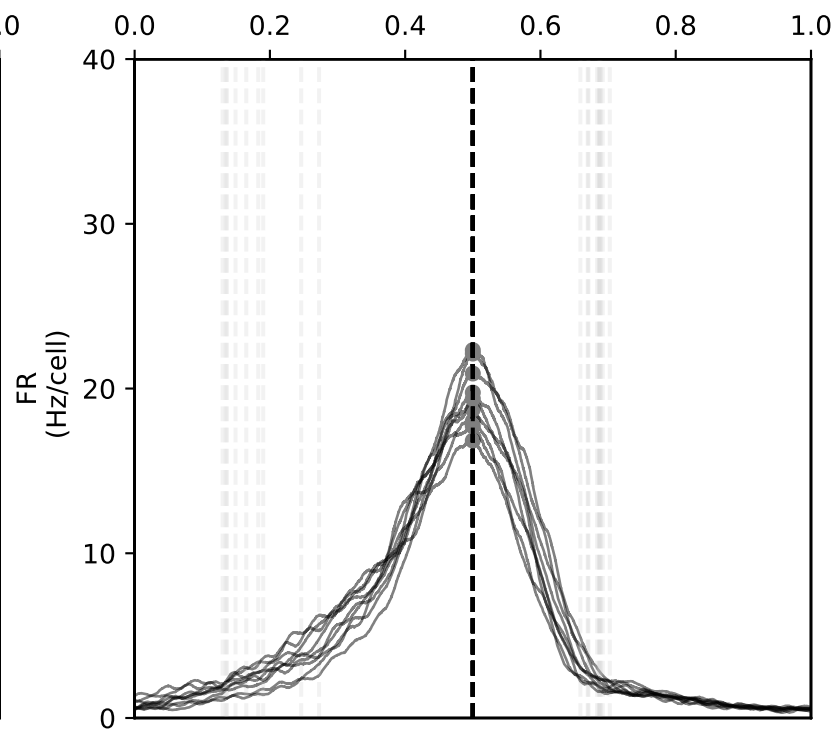

Supplement: Code files — Download Code files, ZIP file. [file eneuro-11-ENEURO.0284-23.2023-s001.zip › prebot-opioid-model-main/figure_notebooks/fig5/fig5_poprate_random_gleak.pdf]

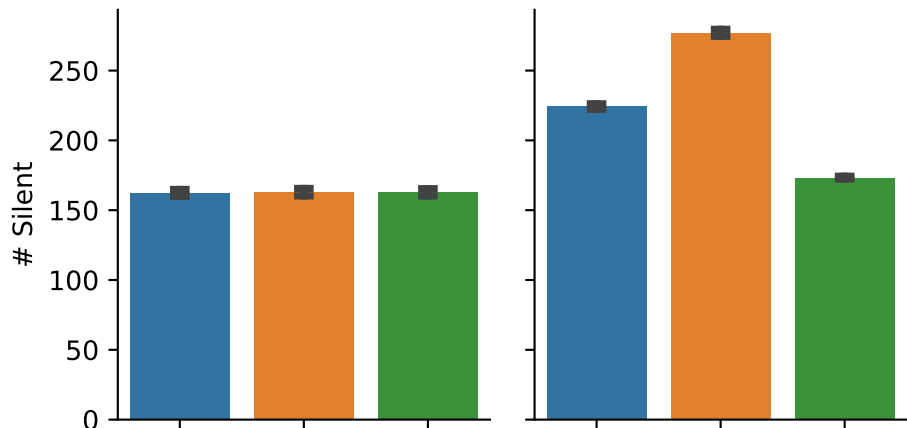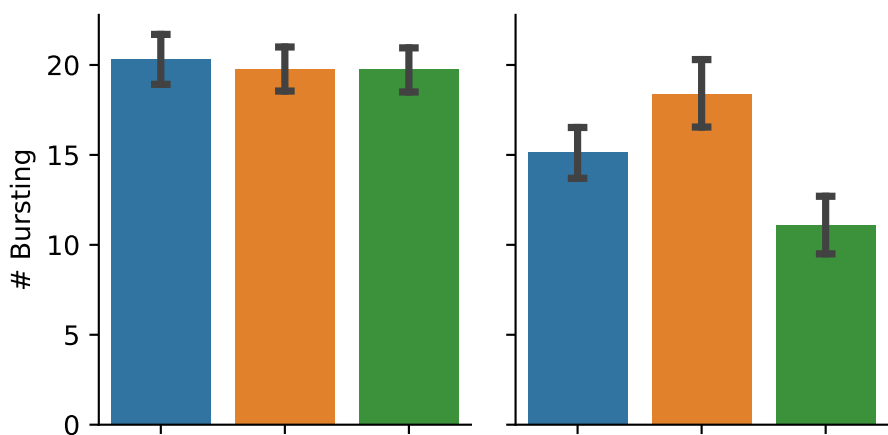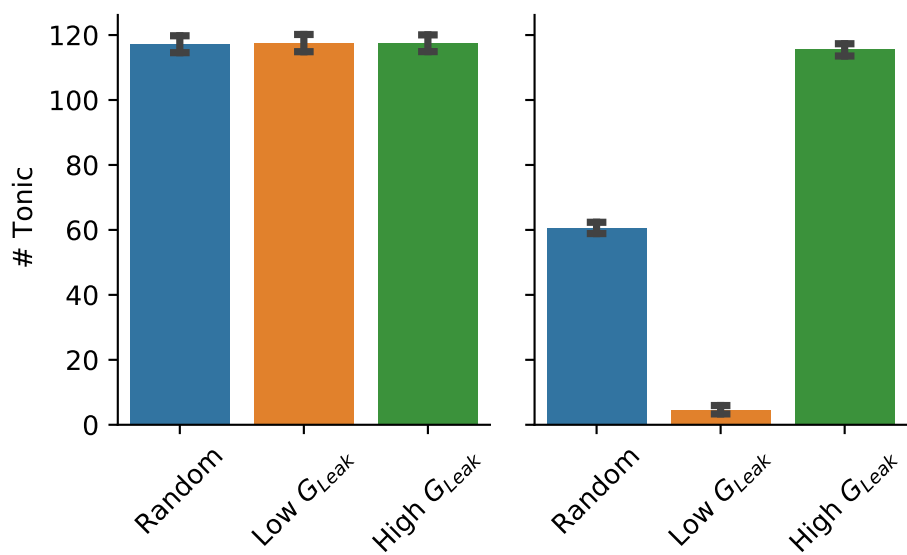

Supplement: Code files — Download Code files, ZIP file. [file eneuro-11-ENEURO.0284-23.2023-s001.zip › prebot-opioid-model-main/figure_notebooks/fig5/fig5_tbq_barplots.pdf]

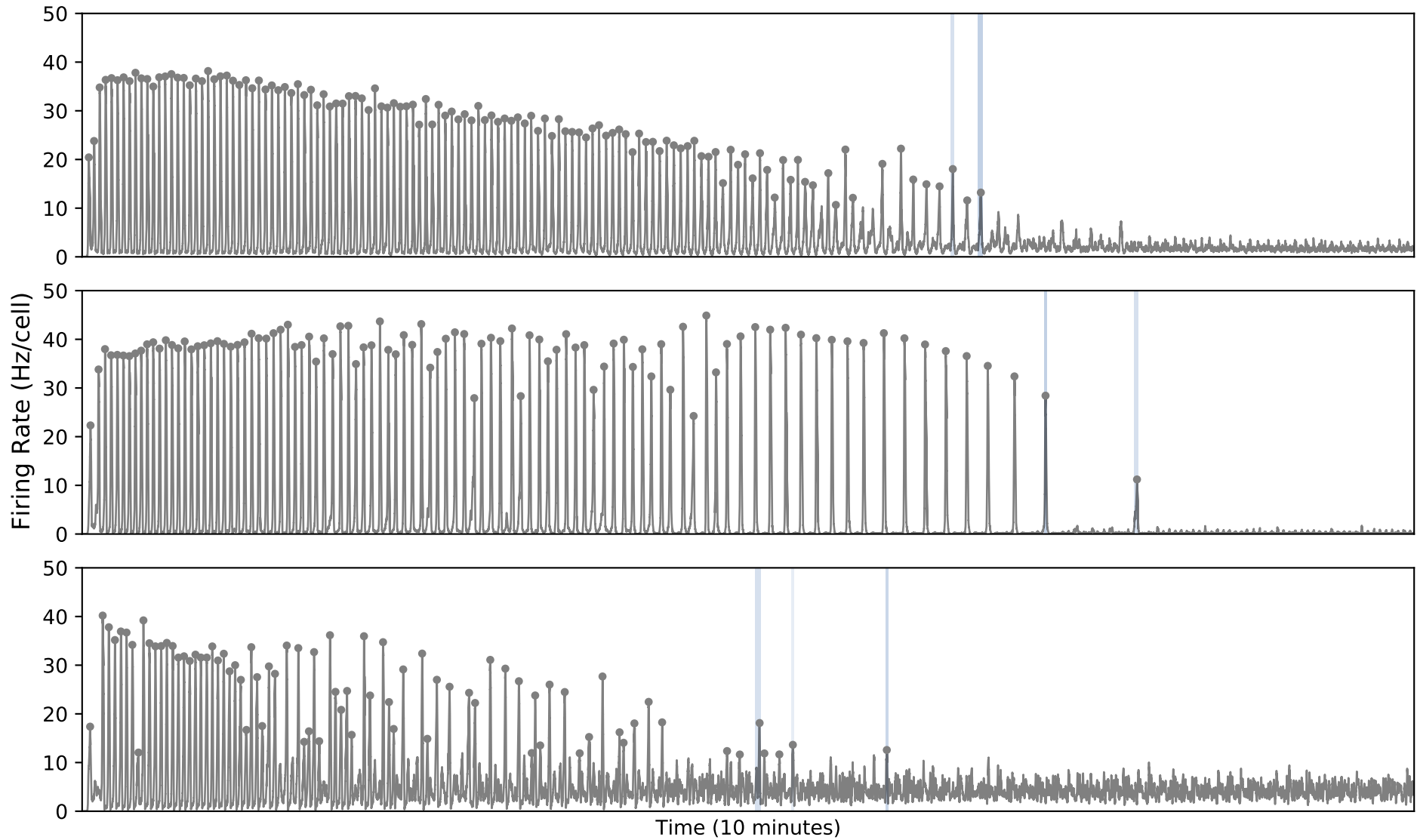

Supplement: Code files — Download Code files, ZIP file. [file eneuro-11-ENEURO.0284-23.2023-s001.zip › prebot-opioid-model-main/figure_notebooks/fig5/fig5_traces.pdf]

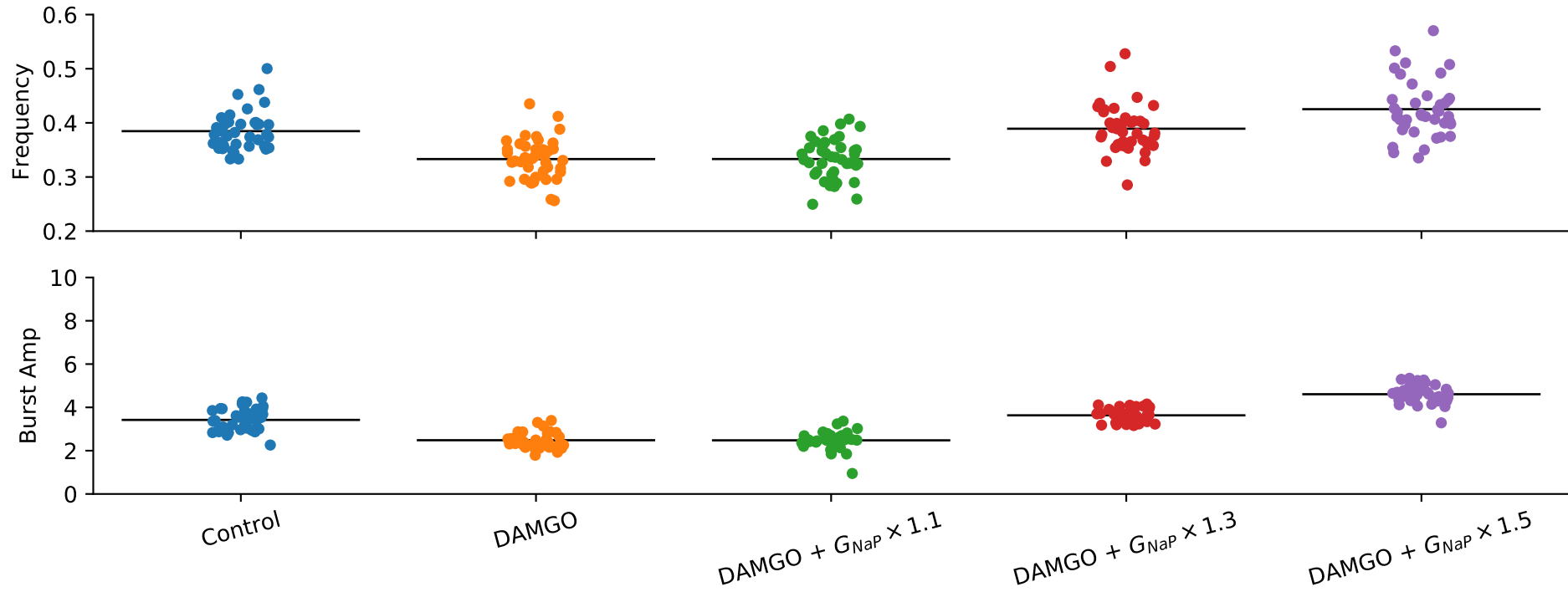

Supplement: Code files — Download Code files, ZIP file. [file eneuro-11-ENEURO.0284-23.2023-s001.zip › prebot-opioid-model-main/figure_notebooks/fig6and7/fig6/fig6_freq_and_amp.pdf]

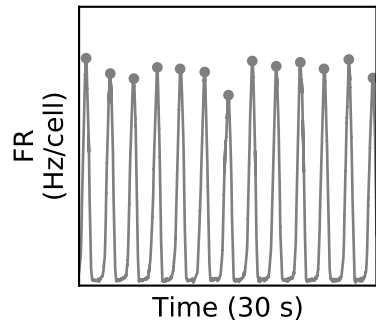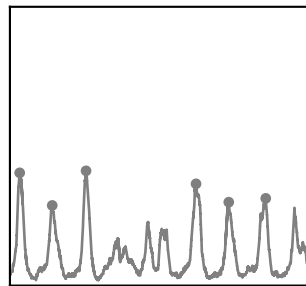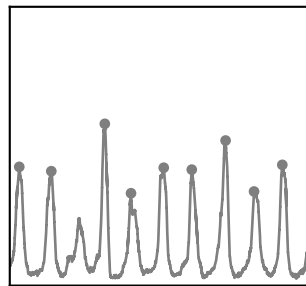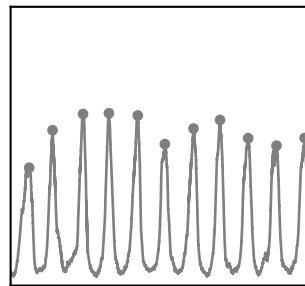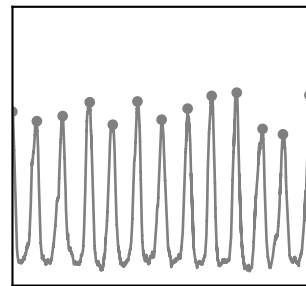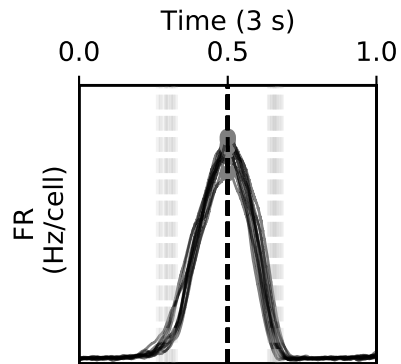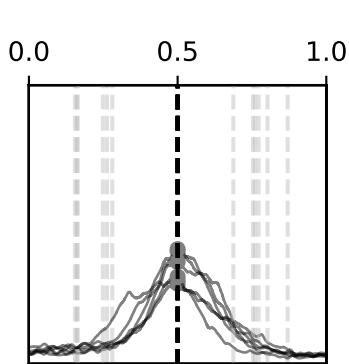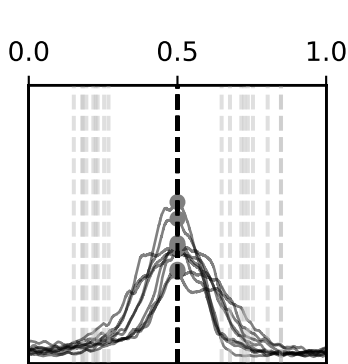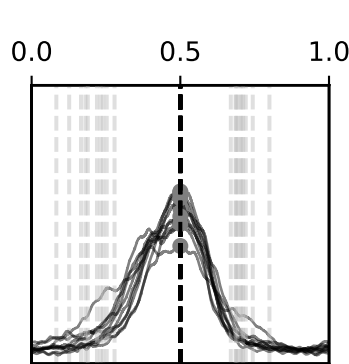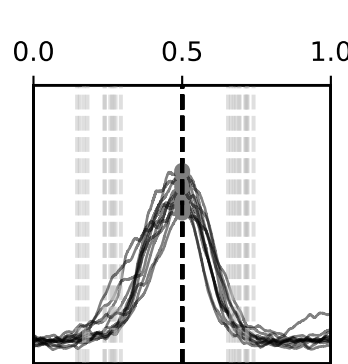

Supplement: Code files — Download Code files, ZIP file. [file eneuro-11-ENEURO.0284-23.2023-s001.zip › prebot-opioid-model-main/figure_notebooks/fig6and7/fig6/fig6_poprate.pdf]

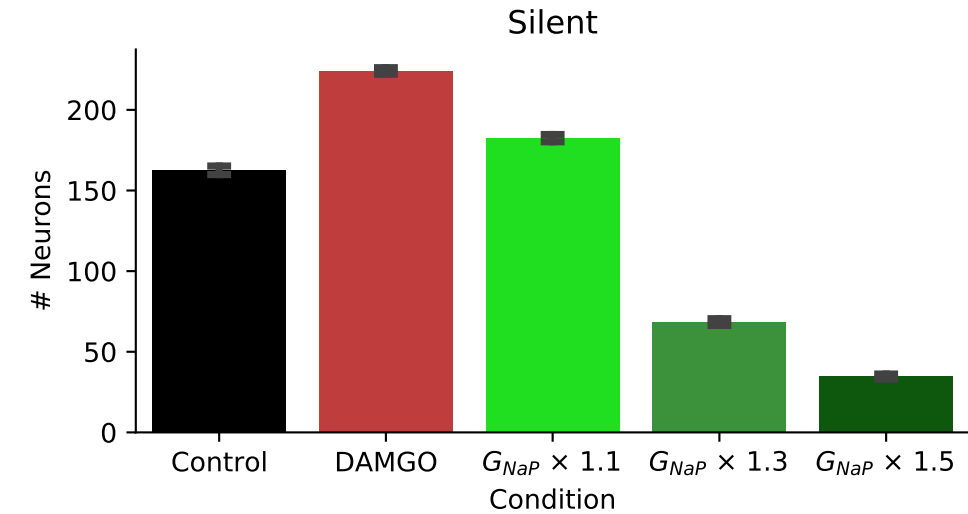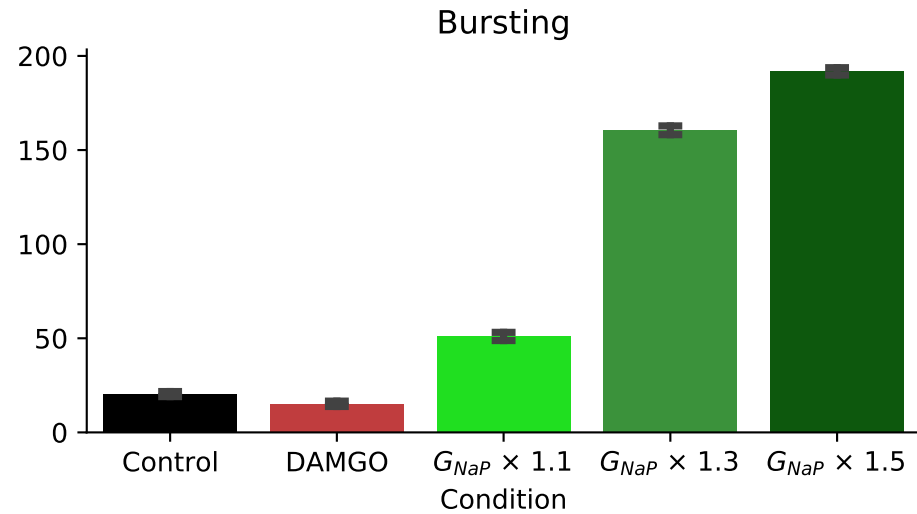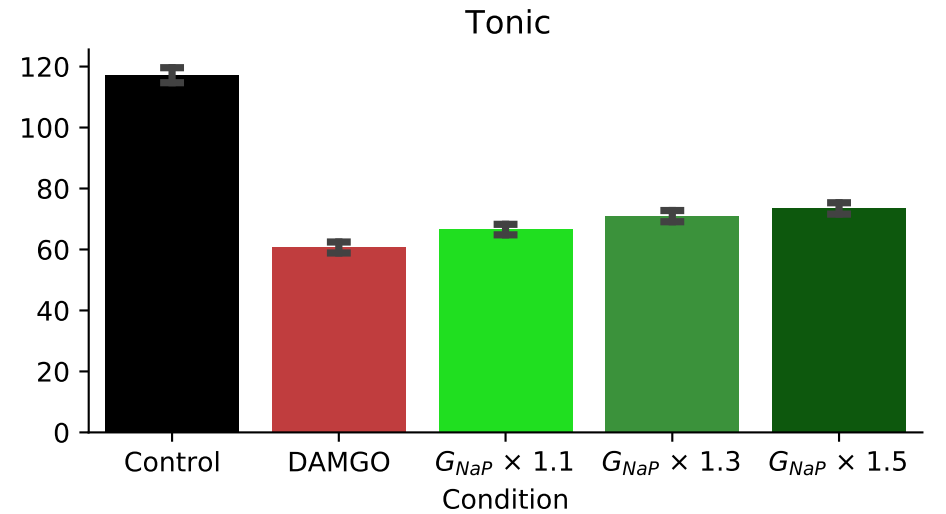

Supplement: Code files — Download Code files, ZIP file. [file eneuro-11-ENEURO.0284-23.2023-s001.zip › prebot-opioid-model-main/figure_notebooks/fig6and7/fig6/fig6_tbq_barplots.pdf]

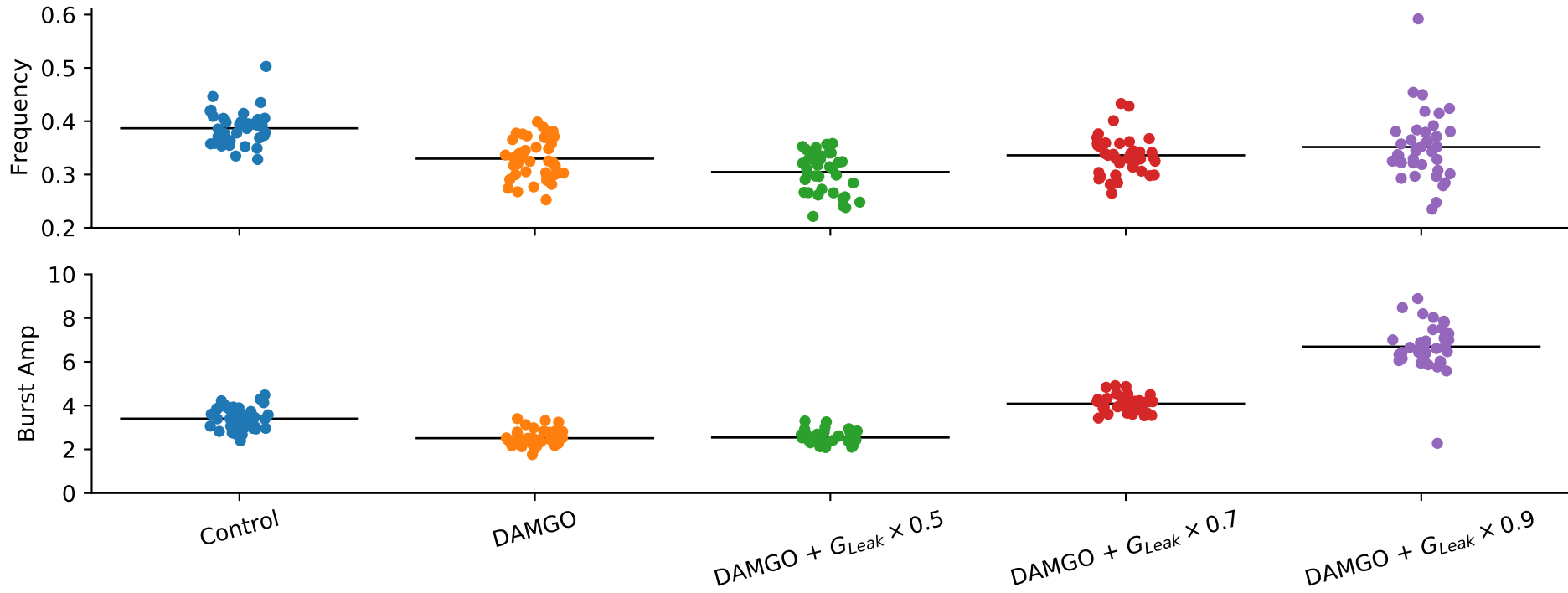

Supplement: Code files — Download Code files, ZIP file. [file eneuro-11-ENEURO.0284-23.2023-s001.zip › prebot-opioid-model-main/figure_notebooks/fig6and7/fig7/fig7_freq_and_amp.pdf]

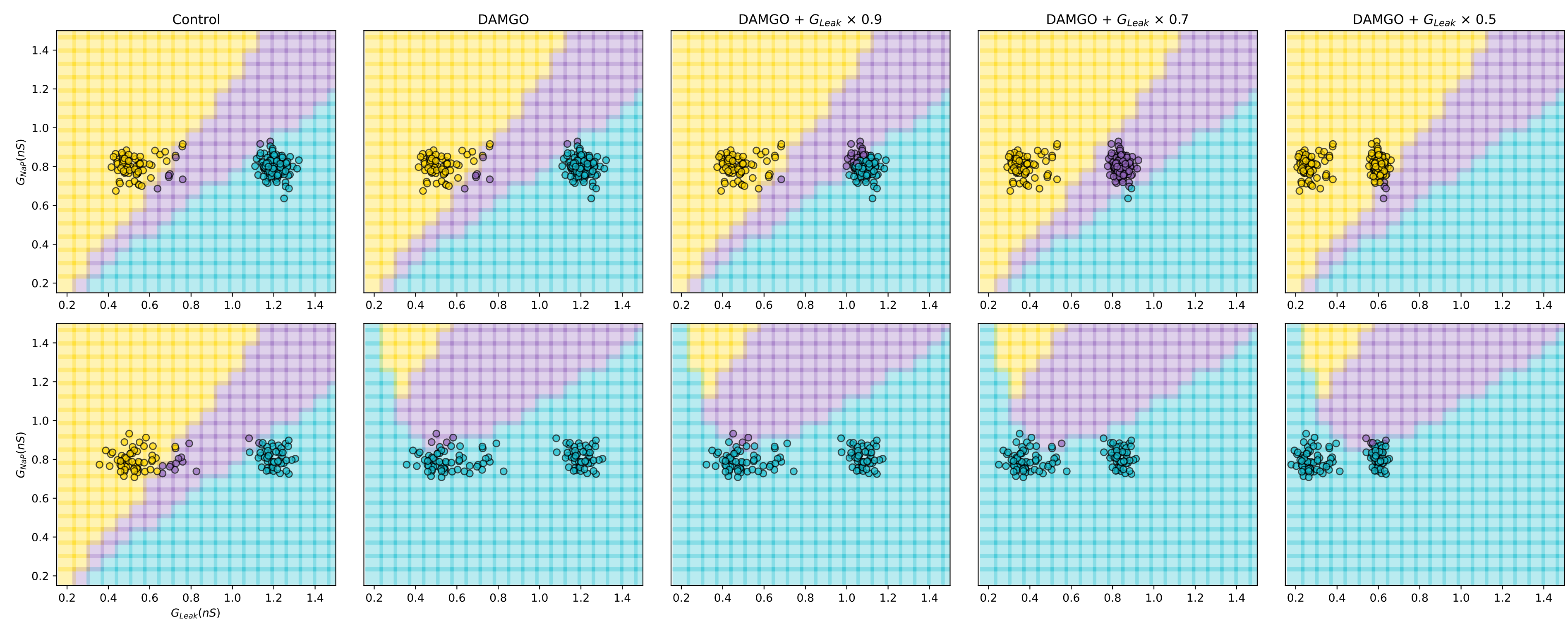

Supplement: Code files — Download Code files, ZIP file. [file eneuro-11-ENEURO.0284-23.2023-s001.zip › prebot-opioid-model-main/figure_notebooks/fig6and7/fig7/fig7_gleak_phase_diagrams.pdf]

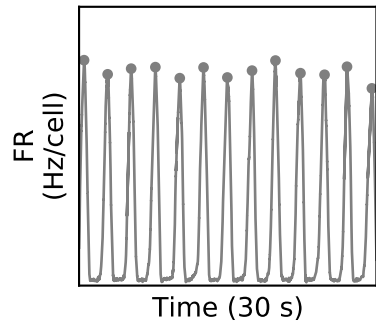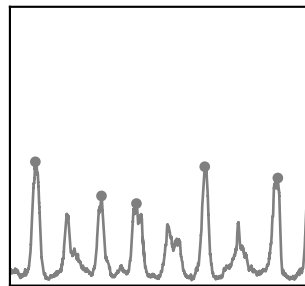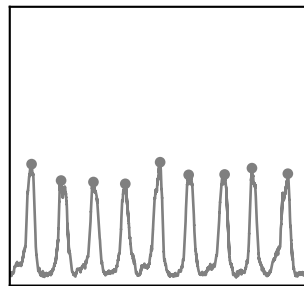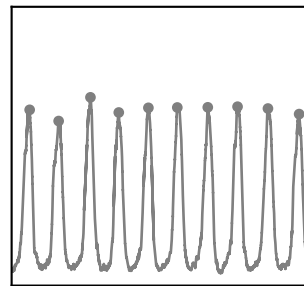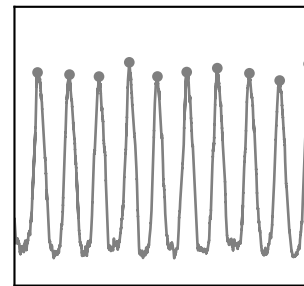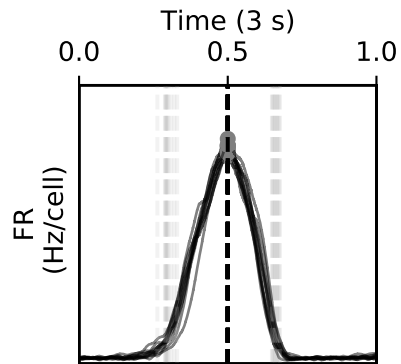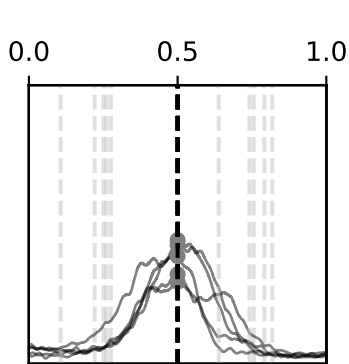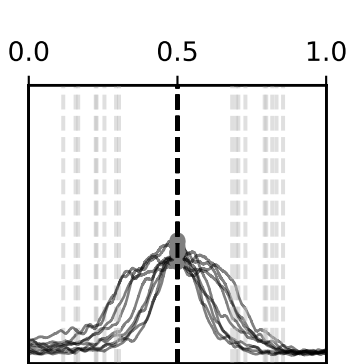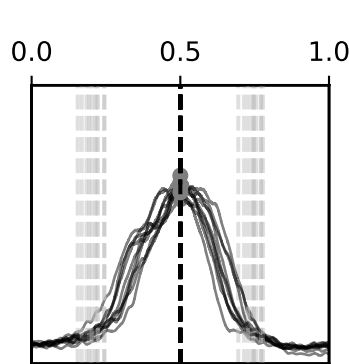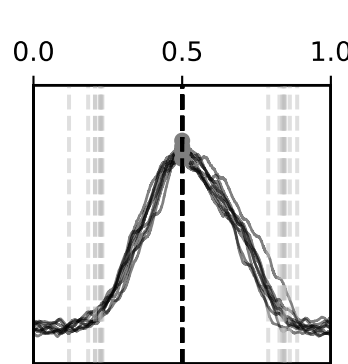

Supplement: Code files — Download Code files, ZIP file. [file eneuro-11-ENEURO.0284-23.2023-s001.zip › prebot-opioid-model-main/figure_notebooks/fig6and7/fig7/fig7_poprate.pdf]

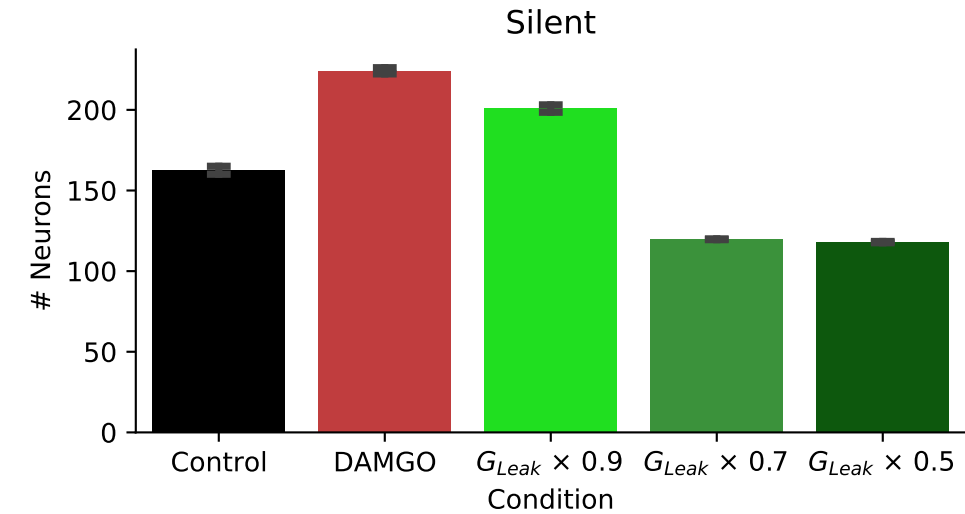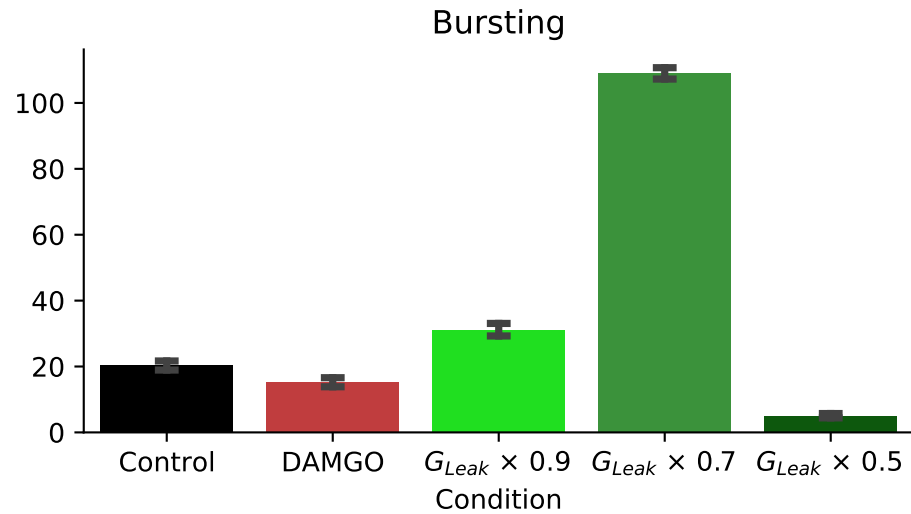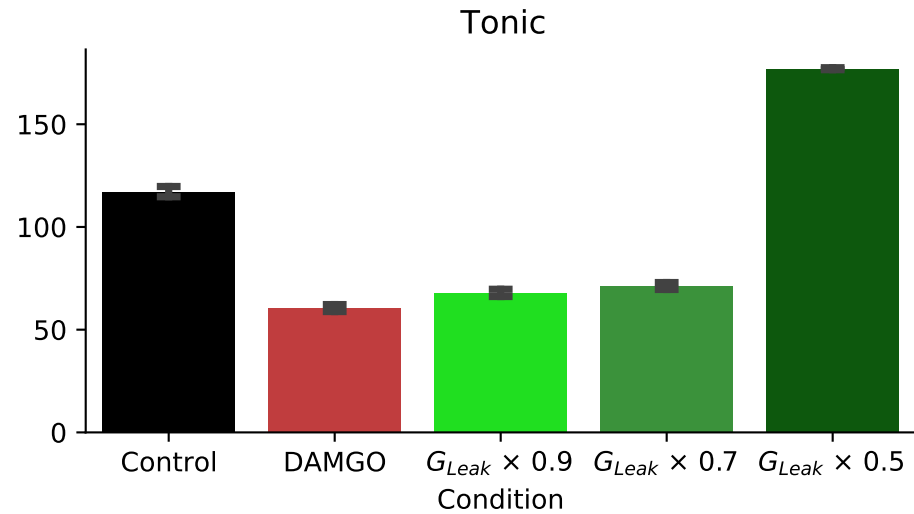

Supplement: Code files — Download Code files, ZIP file. [file eneuro-11-ENEURO.0284-23.2023-s001.zip › prebot-opioid-model-main/figure_notebooks/fig6and7/fig7/fig7_tbq_barplots.pdf]

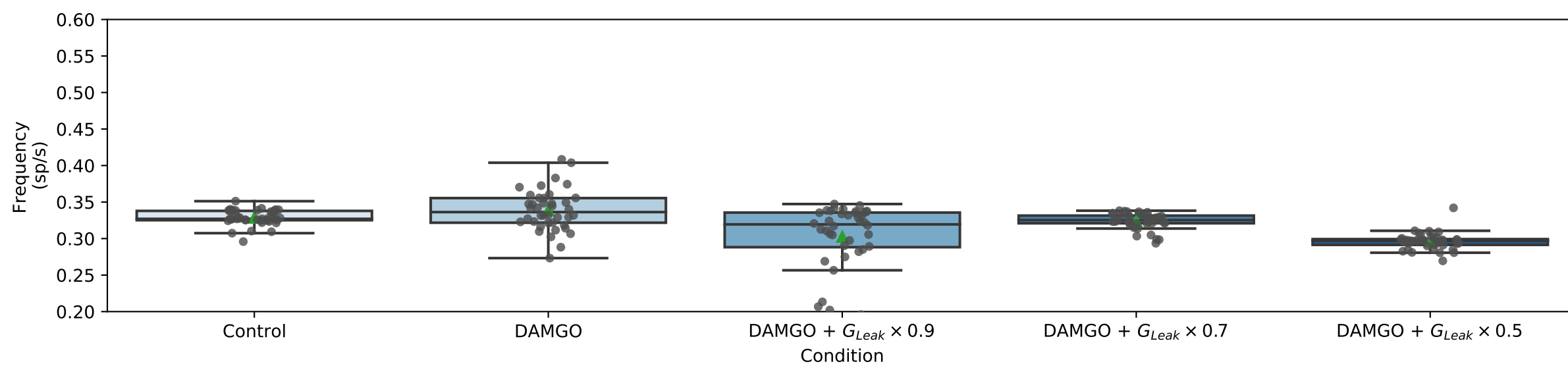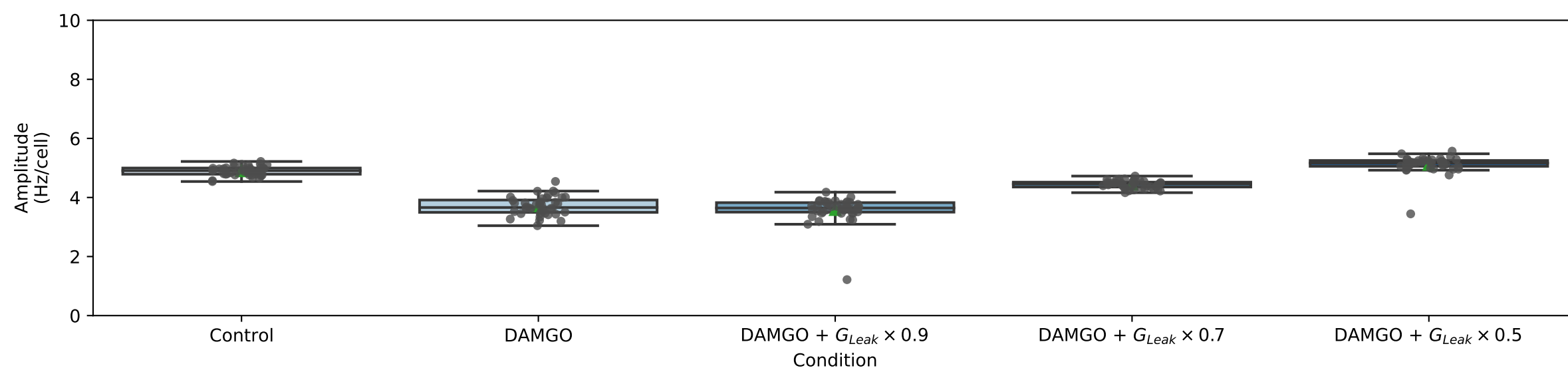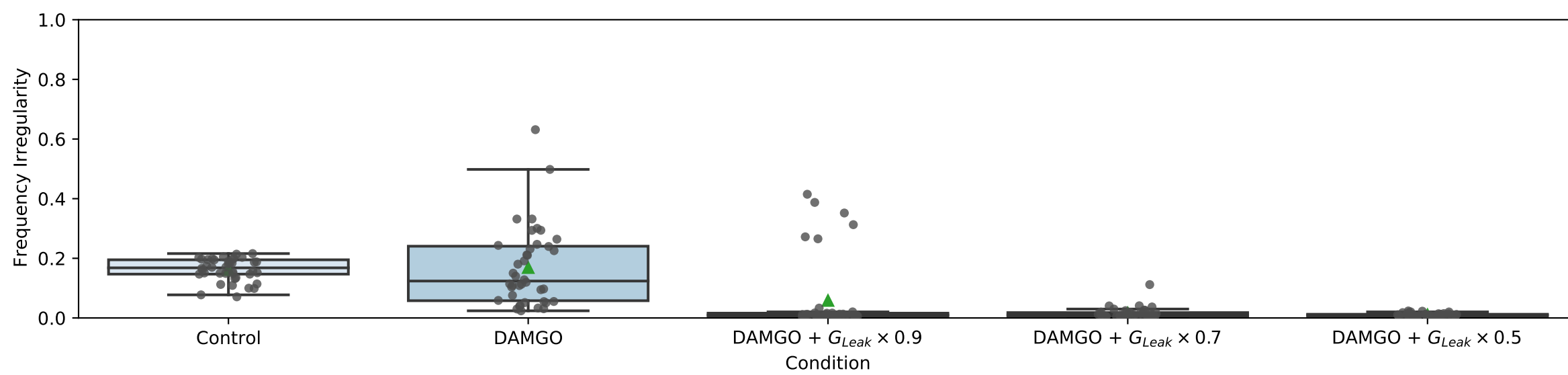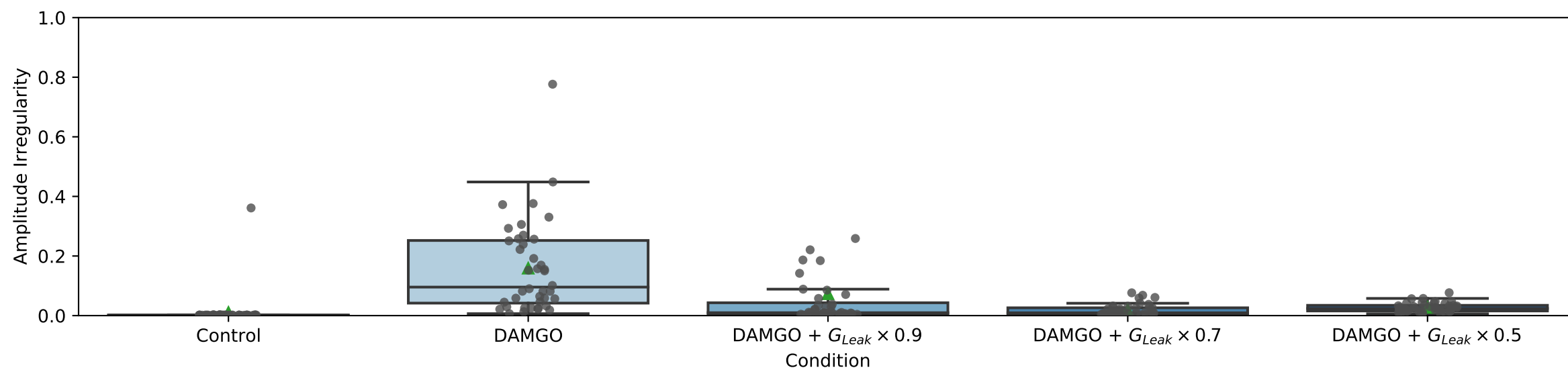

Supplement: Code files — Download Code files, ZIP file. [file eneuro-11-ENEURO.0284-23.2023-s001.zip › prebot-opioid-model-main/simulations/gleak_mod_sim/burst_stats/gleak_dense_boxplots.pdf]

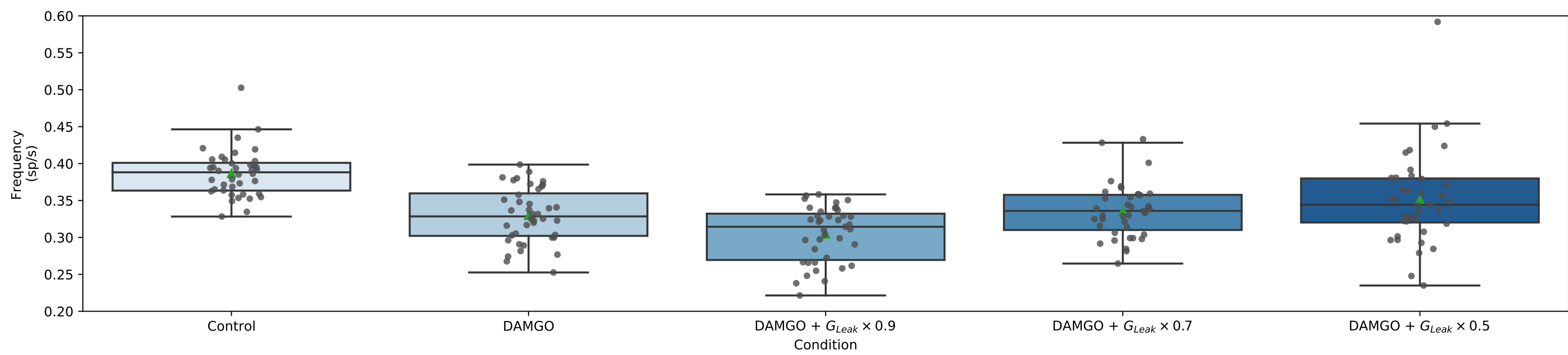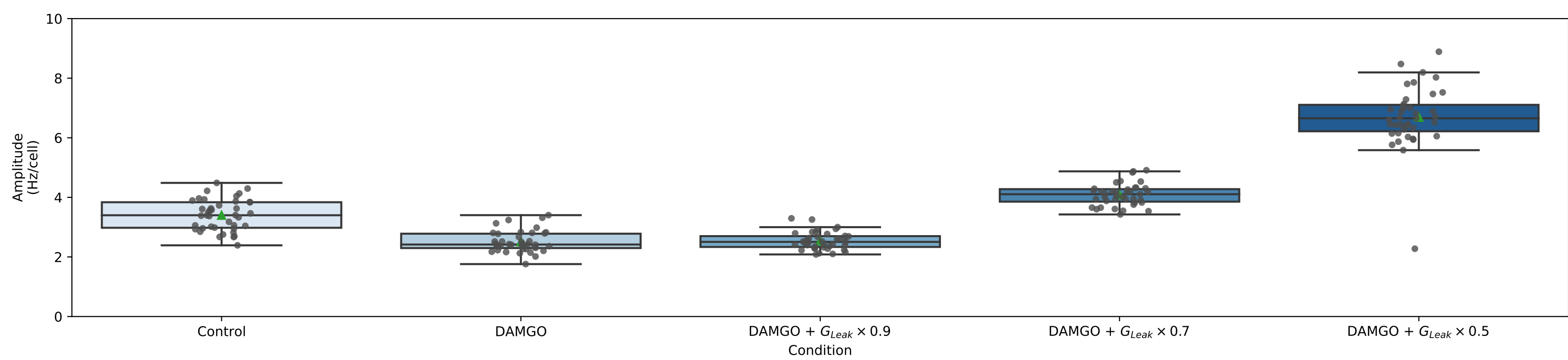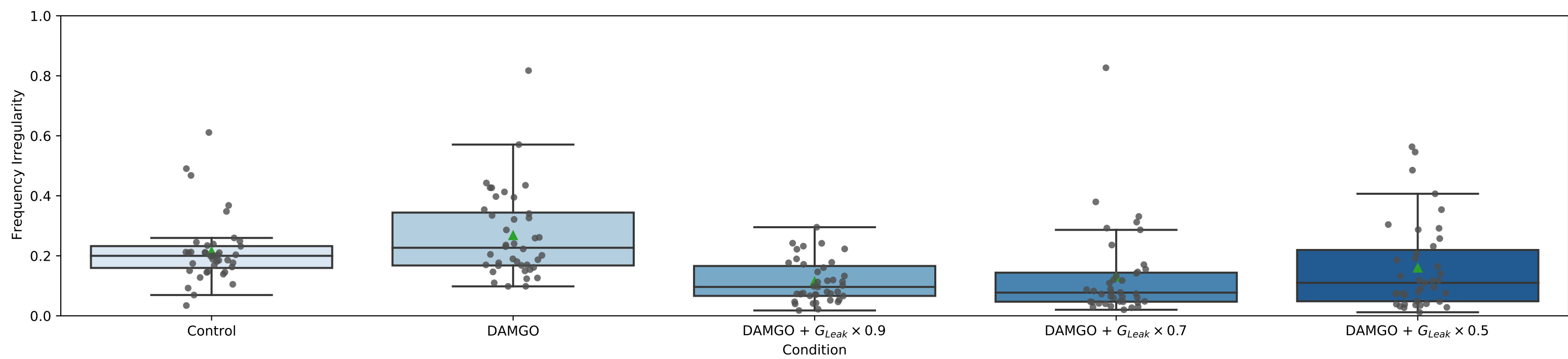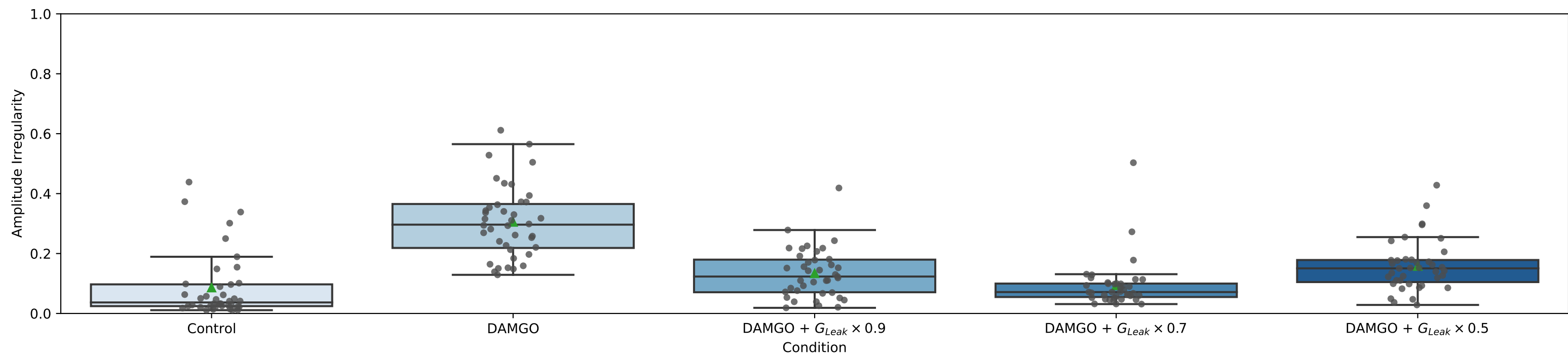

Supplement: Code files — Download Code files, ZIP file. [file eneuro-11-ENEURO.0284-23.2023-s001.zip › prebot-opioid-model-main/simulations/gleak_mod_sim/burst_stats/gleak_sparse_boxplots.pdf]
